# Signalling cognition: the gut microbiota and hypothalamic-pituitary-adrenal axis

**DOI:** 10.3389/fendo.2023.1130689

**Published:** 2023-06-19

**Authors:** Jody A. Rusch, Brian T. Layden, Lara R. Dugas

**Affiliations:** ^1^ Division of Chemical Pathology, Department of Pathology, University of Cape Town, Cape Town, South Africa; ^2^ C17 Chemical Pathology Laboratory, Groote Schuur Hospital, National Health Laboratory Service, Cape Town, South Africa; ^3^ Division of Endocrinology, Diabetes, and Metabolism, Department of Medicine, University of Illinois at Chicago, Chicago, IL, United States; ^4^ Department of Medicine, Jesse Brown Veterans Affairs Medical Center, Chicago, IL, United States; ^5^ Division of Epidemiology and Biostatistics, School of Public Health, University of Cape Town, Cape Town, South Africa; ^6^ Public Health Sciences, Parkinson School of Health Sciences and Public Health, Loyola University Chicago, Maywood, IL, United States

**Keywords:** microbiota-gut-brain axis, hypothalamic-pituitary-adrenal axis, stress, cortisol, glucocorticoids, cognition

## Abstract

Cognitive function in humans depends on the complex and interplay between multiple body systems, including the hypothalamic-pituitary-adrenal (HPA) axis. The gut microbiota, which vastly outnumbers human cells and has a genetic potential that exceeds that of the human genome, plays a crucial role in this interplay. The microbiota-gut-brain (MGB) axis is a bidirectional signalling pathway that operates through neural, endocrine, immune, and metabolic pathways. One of the major neuroendocrine systems responding to stress is the HPA axis which produces glucocorticoids such as cortisol in humans and corticosterone in rodents. Appropriate concentrations of cortisol are essential for normal neurodevelopment and function, as well as cognitive processes such as learning and memory, and studies have shown that microbes modulate the HPA axis throughout life. Stress can significantly impact the MGB axis *via* the HPA axis and other pathways. Animal research has advanced our understanding of these mechanisms and pathways, leading to a paradigm shift in conceptual thinking about the influence of the microbiota on human health and disease. Preclinical and human trials are currently underway to determine how these animal models translate to humans. In this review article, we summarize the current knowledge of the relationship between the gut microbiota, HPA axis, and cognition, and provide an overview of the main findings and conclusions in this broad field.

## 1 Introduction

Humans have a longstanding and intimate, life-long, relationship with microbes, collectively known as the human microbiota, which plays a key role in influencing bodily systems responsible for human health and disease (1–4). The gut microbiota (GM), which comprises a complex, heterogeneous ecosystem of microorganisms, including bacteria, archaea, fungi, protozoa, viruses, and parasites, is a vital component of the human microbiota (5–9). The bacterial population is the most extensively characterised subset of the GM. While initially believed that microbial cells outnumbered human cells by a factor of 10 to 1, the current estimate is approximately 1.3 to 1 (10). Notably, the Human Gut Microbiome (HRGM) has recently been expanded to include 232,098 non-redundant genomes for 5,414 representative prokaryotic species, with over 103 million unique proteins (11). While the human genome is essentially stable and limited in flexibility for the lifespan of the host, the vast genetic potential of the microbes is dynamic and responsive to the environment. This suggests that the GM is an important environmental factor for humans, with evolutionarily conserved roles in the metabolism, immunity, development, and behaviour of the host (2, 12–17). Recently, pivotal roles in endocrine and neural development and function have started to be elucidated (18, 19).

Emerging evidence suggests that the GM, the hypothalamic-pituitary-adrenal (HPA) axis, and cognitive processes are linked bidirectionally *via* multiple pathways, including the vagus nerve, neurotransmitter and metabolite production, immune system and blood-brain barrier regulation, and hormone metabolism. Alterations in the GM, whether due to diet, antibiotics, or other factors, can impact the stress response, HPA axis activity, and overall cognitive health. This review aims to summarize the current understanding of the role of the GM in regulating the HPA axis, a key component of the gut-brain axis. The review further explores the mechanisms and pathways through which the GM can alter gut-brain communication, with an emphasis on the effects on the HPA axis and its influence on cognition.

## 2 The microbiota-gut-brain axis

The MGB axis refers to the bidirectional communication network between the CNS, the autonomic nervous system (ANS), the endocrine system, the immune system, and the GM (20–23). This system enables microbes to share information with the brain, and the brain to communicate with the gut (24, 25). Despite extensive data from animal and human studies supporting the role of the GM in the MGB axis, the mechanisms by which the GM impacts the human brain are not yet fully understood. What is known has primarily been learnt from studies using germ-free animal models, and studies examining the effects of specific microbial species, probiotics, antibiotics, and infections. Further, technological advancements in sequencing and metabolomics have enabled scientists to explore this topic more thoroughly.

As a key regulator, the GM can modulate host physiological processes through several proposed mechanisms (25, 26). These include microbial constituents [e.g. lipopolysaccharides (LPS) and peptidoglycans (PG)] (25), microbial products [e.g. enzymes, short chain fatty acids (SCFAs), and neurotransmitters] (27, 28), hormone release (e.g. glucocorticoids) (29), and substrate metabolism [e.g. bile acids (BAs) and tryptophan] (30, 31). In the gut, the GM and its metabolites have been shown to modulate gastrointestinal functions *via* their effects on intestinal permeability (32–35), mucosal immune function (36–38), intestinal motility and sensitivity (39, 40), and the enteric nervous system (ENS) (21, 24, 25, 41). The GM can stimulate the release of peptides and hormones from enteroendocrine cells, which can have direct or indirect central effects (22, 39). Further neuroendocrine and metabolic pathways link the GM with the CNS (21).

The GM is critical to the development and functioning of the CNS. Studies in animals have demonstrated the effect that the GM has on neural development and neurochemistry in the host, influencing the stress system, behavior, and cognition (42–47). Conversely, the brain can affect intestinal function and the GM, for example, by HPA axis-mediated glucocorticoid modification of immunity in response to stress (29).

### 2.1 The role of the HPA axis in the MGB axis

The HPA axis plays a central role in mediating the stress response and regulating the interaction between the GM, gut, and brain (42).

Mechanistically, cortisol can impact the MGB axis through multiple pathways. Cortisol receptors are expressed on various cells of the gut, including epithelial cells, immune cells, and enteroendocrine cells, indicating a direct effect of cortisol on gut function (21, 29, 48). Cortisol can also affect the gut microbiota by altering gut transit time, intestinal permeability, and nutrient availability, which can in turn impact the composition and diversity of the GM (48). Furthermore, cortisol can impact the brain by binding to glucocorticoid receptors (GRs) located in various brain regions, including the hippocampus, amygdala, and prefrontal cortex. There is also evidence for signaling between the GM and CNS, since microbes residing in the gut can activate stress circuits in the CNS through the vagus nerve and sensory neurons of the ENS (21, 49–54).

Chronic or prolonged stress can lead to dysregulation of the HPA axis, which can have negative effects on various bodily systems, including the MGB axis (55). Elevated cortisol levels have been associated with alterations in GM composition and increased gut permeability, which can lead to inflammation and contribute to brain dysfunction and various CNS disorders (56).

The effects of dysregulation of the HPA axis has primarily been studied using various modalities of stress. Preclinical evidence suggests that GM-mediated mechanisms are likely involved in modulating brain processes, including brain biochemistry, response to stress, pain interpretation, feeding, emotional behaviors, and cognition (57–64). Initial clinical evidence of MGB interactions primarily stems from associations between dysbiosis and CNS disorders, such as autism, anxiety-depressive behaviors, and functional gastrointestinal disorders (63, 65–72). Furthermore, recent studies have shown dramatic changes in the GM of patients with Alzheimer’s disease, Parkinson’s disease, multiple sclerosis, and schizophrenia (73–77). Fecal microbiota transplant (FMT) studies have added evidence for causality by inducing many of the symptoms of these diseases in germ-free animals (78).

### 2.2 Development and life course of the MGB axis

There exists some controversy regarding prenatal exposure to microbes and their importance to fetal development (79, 80). However, the acquisition of microbes occurs primarily at birth, with delivery through the birth canal exposing the infant to its mother’s microbiota, resulting in vertical transmission of an initial maternal signature (81). Caesarean section delivery alters the initial microbial composition (82). After birth, several factors influence GM composition in early life, including breastfeeding, nutrition, infection and antibiotic use, environmental stressors, and host genetics (83). While initial microbial diversity is low, it escalates rapidly as a function of diet and environment, with an increase in the relative composition of strict anaerobes (84). A more stable and complex, adult-like microbiota starts to emerge as early as one to three years of age, although this development may continue as late as pre-adolescence (85, 86). The first year of life encompasses a critical “window period” of development in which the GM may be more susceptible to environmental influences and highly influential with regards to the overall health of the host. This critical window of GM development aligns with critical windows of development of other systems that are also more sensitive and vulnerable to environmental input, such as the immune system, HPA axis, and brain development in general.

The GM of children is characterized by relatively higher abundances of microbes with genes that function to support human development, including vitamin biosynthesis and polysaccharide and xenobiotic metabolism (84). Adolescence is characterized by an intense period of sexual development and growth, and recent data indicate that the GM undergoes progressive changes during this period likely due to hormonal surges, stressors, and other age-related factors (87). In adulthood, the core GM is relatively stable, and the main factors influencing its composition are diet, exercise, stress, disease, and medications (88). Confirming the role of the GM in cognitive function in midlife, Meyer et al. explored the relationship between gut microbial community composition and cognitive function in 597 middle-aged adults, and found a significant positive association between β-diversity and all higher cognitive functions tested (89).

The aging process of the host parallels a continuous aging progression of the GM (90). Stability begins to decline as we age, and studies suggest that there is greater inter-individual variation in older adults than younger adults (<65 years old) (91, 92) There is also evidence that the diversity levels of the GM differ with aging, with a reduction in the number of different bacterial species present and changes in the relative abundance of different bacterial phyla, with a decrease in beneficial bacteria such as Bifidobacteria and an increase in potentially harmful bacteria such as Proteobacteria (93–96). Microbial composition can predict human chronological age relatively accurately (97). In a study that used metagenomic profiles from over 4,000 healthy people aged 18–90, the authors were able to construct an algorithm that predicted individuals’ ages within approximately six years of their actual age (98). Functionally, an associated shift towards a more pro-inflammatory state has been noted, along with a decline in immune function, making older individuals more susceptible to infections and other diseases (96, 99). The GM of older individuals may be less efficient at metabolizing certain nutrients, which could affect overall health (92). These alterations may contribute to age-related inflammation, oxidative stress, and neurodegeneration, and have been associated with age-related health issues such as frailty and cognitive decline (92, 100, 101).

There is growing evidence that age-related changes in the microbiota may contribute to cognitive decline and other neurodegenerative disorders (102, 103). Human studies comparing the composition of the GM of elderly participants suffering from cognitive impairment with healthy individuals indicate significant changes in GM composition. Specifically, there is an increase in pro-inflammatory taxa and a reduction in anti-inflammatory taxa (104–109).

The HPA axis also undergoes age-related changes including alterations in the sensitivity and responsiveness of the hypothalamus and pituitary gland to negative feedback by cortisol, changes in the levels and circadian rhythm of circulating cortisol, changes in the expression and function of CRH and glucocorticoid receptors in the hypothalamus, hippocampus, and prefrontal cortex, changes in the activity and connectivity of brain regions involved in stress regulation, including the amygdala, hippocampus, and prefrontal cortex, and changes in the microbiota, which can impact HPA axis function and contribute to inflammation and oxidative stress (110). Essentially, the HPA axis appears to become less responsive with aging, contributing to the dysregulation of stress responses and the resultant development of cognitive decline (111, 112).

Similar to aging, dysbiosis has been associated with increased cortisol secretion, decreased negative feedback at the level of the hypothalamus, and changes in the circadian rhythm of cortisol release (113).

### 2.3 The influence of the gut microbiota on neurodevelopment

Animal studies suggest that the GM plays a crucial role in regulating early brain development (19, 45, 60, 114–117). During key prenatal and postnatal periods, neurodevelopment depends on the integration of environmental cues, such as MGB axis molecular signaling (116). The maturation of neuronal networks is essential for the developing nervous system to form functional neural circuitry. Microglial cells, innate immune cells of the brain, play a critical role in the elimination of unnecessary synaptic connections required for this maturation process (118, 119). The GM appears to influence microglial development and maturation (120, 121), and animal models have demonstrated downstream effects on various aspects of neurocognitive function (19, 45, 60, 114, 115). In contrast, early-life disruptions of gut colonization have been linked to CNS alterations (122).

Germ-free animal models have been crucial in developing an understanding of the role that the GM plays in neurodevelopment (123). The nervous systems of germ-free animals develop differently from those conventionally colonized, exhibiting key differences in multiple neurotransmitter systems and their receptors (25). They display increases in neurogenesis, hippocampal and amygdala volume, myelination, and myelin plasticity-related genes in the prefrontal cortex (25). Their dendrites are longer, and spines are denser, but there are fewer synaptic connections. In the amygdala, they have increased synaptic and neural plasticity-related genes and increased neuronal activity-related genes (124). They present with immature microglia and decreased immune system-related genes. Due to the decreased expression of tight junction proteins, they have a more permeable blood-brain barrier (BBB). Correspondingly, they have impaired immune systems, dysregulated hormone signalling, altered metabolism, and differences in neurotransmission (25). Finally, germ-free animal studies demonstrate that the GM is necessary for normal stress responsivity, anxiety-like behaviors, sociability, and cognition (123).

## 3 Mechanisms and pathways of the MGB axis

**Figure 1:**
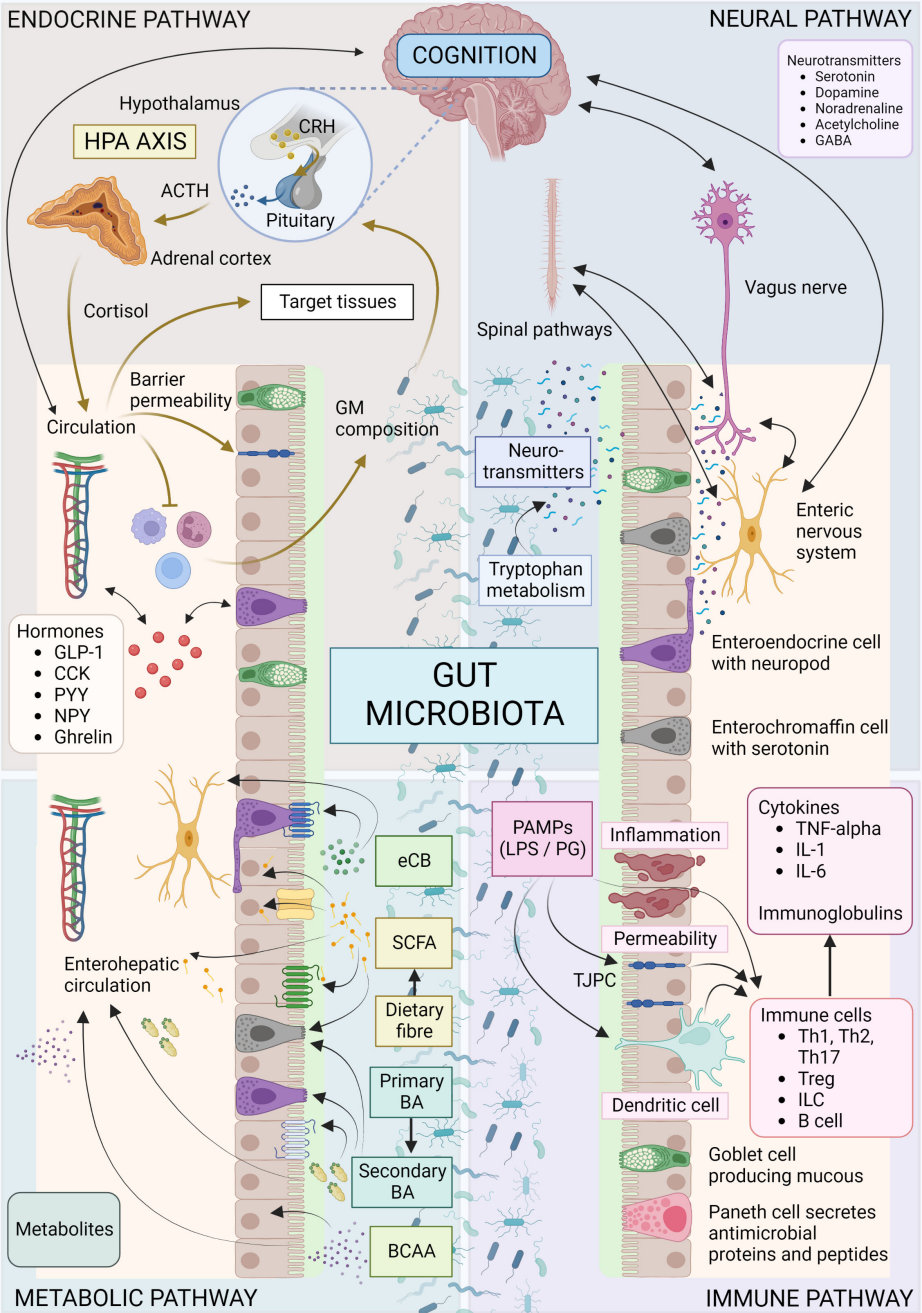
Overview of microbiota-gut-brain axis. Bidirectional communication mechanisms of the MGB axis include endocrine, neural, metabolic and immune system pathways. The hypothalamic-pituitary-adrenal axis is a major neuro-endocrine system responding to stress with the release of corticotrophin-releasing hormone (CRH) from the hypothalamus, and the subsequent release of ACTH from the pituitary, then cortisol from the adrenal cortex. Cortisol reaches target tissues through the circulation, modulates the immune system, and impacts on GM composition and gut permeability. The GM in turn is able to influence the stress response (for e.g., the HPA axis can be activated in response to increased circulating cytokines subsequent to bacterial translocation). Various GM and enteroendocrine cell interactions result in the release of hormones that work locally or on target tissues such as the brain, *via* the circulation. The vagus nerve, enteric nervous system, and spinal pathways provide rapid neural communication routes, while neurotransmitters or their precursors can be produced or metabolized by microbes. Metabolites such as SCFA, BA and eCB may be produced or modified by microbes and bind specific cell receptors in the gut or they may be absorbed into circulation and affect target tissues. Microbes and their products may interact with the immune cells with downstream pro-inflammatory or anti-inflammatory effects. ACTH, Adrenocorticotropic hormone; BA, bile acid; BCAA, branched chain amino acids; CCK, cholecystokinin; CRH, corticotropin-releasing hormone; eCB, endocannabinoid; GABA, γ-aminobutyric acid; GLP-1, glucagon-like peptide 1; GM, gut microbiota; HPA, hypothalamic-adrenal-pituitary; IL, interleukin; ILC, innate lymphoid cells; LPS, lipopolysaccharide; PYY, Peptide YY; NPY, neuropeptide Y; PAMP, Pathogen-associated molecular pattern; PG, peptidoglycan; SCFA, short chain fatty acid; Th, T helper cell; TJPC, tight junction protein complex; T reg, regulatory T cell; TNF-α, tumor necrosis factor-α. Figure created with BioRender.com.

Although the precise mechanisms involved in the crosstalk between the GM and brain remain to be fully determined, there are several putative mechanisms and pathways (125). Microbes influence CNS processes *via* modulation of the nervous system (52), endocrine system (51, 126), and immune system (120), together with their ability to synthesize neurotransmitters (127–129) and produce metabolites (127, 130–132) (**Figure 1**). Together, these mechanisms and pathways illustrate the complex interplay between the GM and the brain, highlighting the importance of understanding the MGB axis in health and disease.

### 3.1 The HPA axis, glucocorticoids, and the stress response

The HPA axis is a major stress response system in the body, with neurons in the hypothalamic paraventricular nucleus (PVN) synthesizing and secreting corticotropin-releasing hormone (CRH) and antidiuretic hormone (ADH) in response to stress (133). These peptides stimulate the release of adrenocorticotropic hormone (ACTH) from corticotropic cells of the anterior pituitary gland, which in turn targets the zona fasciculata of the adrenal cortex to produce glucocorticoids, such as cortisol in humans and corticosterone in rodents (133–135). Cortisol acts on the hypothalamus and pituitary in a negative feedback loop to regulate the response (133). The PVN’s activity is regulated by various afferent systems, including the sympathetic (SNS) and parasympathetic nervous system (PNS), and limbic circuits (136). The interplay of central and peripheral systems produces the characteristic behavioral, endocrine, autonomic, and immune responses to stress (135, 137). The release of cortisol is characterized by both circadian and 60- to 90-minute oscillations (138). The normal 24-hour profiles of both ACTH and cortisol show an early morning peak, decreasing concentrations throughout the day, a nadir around midnight, and an abrupt elevation during late sleep culminating in the early morning peak (138).

Glucocorticoids are essential for regulating cellular processes, including metabolism, growth, differentiation, and apoptosis, and act *via* intracellular receptors in the nuclear receptor superfamily (139). They regulate the transcription of target genes in organ systems that maintain homeostasis and help the body cope with physical and psychological stress (134, 135). Glucocorticoids are involved in several processes related to host defense, including immunity and inflammation, as well as metabolism, growth, cardiovascular function, water and electrolyte balance, reproduction, and mood and cognition (49, 140–148).

The brain is a crucial target organ for glucocorticoids, and their actions are mediated by the mineralocorticoid receptor (MR) and GR, which act as transcription factors and mediate non-genomic steroid effects (149). During the early stages of acute stress, MR activation is required for the appraisal process and memory retrieval, while GR promotes memory consolidation and behavioral adaptation (150). Glucocorticoids also play a vital role in central nervous system (CNS) development and are required for normal maturation (139). In adulthood, they contribute to neuronal plasticity, and have been implicated in neurodegenerative processes.

HPA axis dysregulation can result in hyper- or hypocortisolism, excessive or dampened reactivity to stressors, and circadian rhythm abnormalities (17). HPA axis dysfunction is linked to a decline in cognitive function, aging, immune system dysfunction, and systemic inflammation (135). Individuals with altered HPA axis function are also more likely to develop metabolic disorders such as cardiovascular disease, diabetes, and inflammatory bowel disease (IBD) (142, 151). Neuropsychiatric symptoms such as depression, mania, anxiety, and neurocognitive impairment are associated with both hyper- and hypocortisolism (143, 144). HPA axis dysfunction is also associated with many major psychopathologies, including autism, anxiety disorders, depression, and schizophrenia, as well as other cognitive disorders (145).

### 3.2 The nervous system

#### 3.2.1 The autonomic nervous system

The ANS regulates involuntary physiological processes throughout the body, except for skeletal muscle, providing neural control (152). In the gastrointestinal system, both the SNS and PNS transmit afferent signals arising from the lumen to the CNS (via enteric, spinal and vagal pathways) and efferent signals from the CNS to the intestinal structures (153). The PNS, which includes the vagus nerve, provides both excitatory and inhibitory control over gastric, intestinal, and pancreatic functions (154). On the other hand, the SNS, predominantly inhibits gastrointestinal muscle and mucosal secretion and regulates blood flow through neural-dependent vasoconstriction. The ENS is the third and largest component of the ANS. The individual components of the MGB axis communicate bidirectionally within the ANS. Additionally, in combination with the HPA axis and neuroendocrine signaling, the ANS can induce CNS-modulated changes in the gut (155).

##### 3.2.1.1 The enteric nervous system

The ENS is a mesh-like system of 200 to 600 million neurons embedded in the gastrointestinal system’s lining, which facilitates communication between the brain and the GM (53). The ENS has several functions, including food propulsion, nutrient handling, blood flow regulation, and immunological defense (37, 153, 156). It is crucial in maintaining homeostasis and a stable gut microenvironment, in collaboration with the intestinal immune system, endocrine system, and the GM (53). Structurally, it is arranged into two ganglionated plexuses, the submucosal plexus (Meissner’s plexus) and the myenteric plexus (Auerbach’s Plexus), consisting of nitrergic (nitric oxide-dependent) and cholinergic (acetylcholine-dependent) enteric neurons (53).

The ENS can independently manage gastrointestinal function since it is equipped with intrinsic reflex microcircuits (157, 158). Moreover, the ENS produces more than 30 neurotransmitters, and the hormones and peptides it releases into circulation can cross the blood-brain barrier (BBB) and synergistically act with the vagus nerve (159). Neuropod cells, a recently discovered type of enteroendocrine cell, can transduce signals from the ENS to sensory neurons (160). There are many commonalities between the ENS and the CNS in terms of neurotransmitters, signaling pathways, and anatomical properties (53), which is why the ENS is referred to as the “little brain” (161). Although the ENS provides independent control over gastrointestinal function, the CNS provides extrinsic neural inputs that modulate, regulate and integrate these functions *via* the vagus nerve, thoracolumbar, and lumbosacral spinal cord (154).

Interactions between the GM and ENS have garnered significant attention in the past decade. Cooperative interactions between the ENS, GM, and intestinal immune and endocrine systems maintain host homeostasis (53). The GM can influence the development and function of the ENS directly and indirectly due to close proximity. For instance, early exposure to intestinal microbes is crucial for the postnatal development and organization of the ENS (53). Germ-free mice display abnormalities in ENS structure, such as reduced enteric neurons, with associated deficits in gut motility (53). They also exhibit attenuated intrinsic sensory signaling, defective influx of enteric glial cells into the intestinal mucosa, and altered neurochemistry (114, 162, 163).

Enterochromaffin cells of the ENS are another intermediary that facilitates communication with the GM (164). The biosynthesis of serotonin by enterochromaffin cells is promoted and enhanced by the GM, and is necessary for mucosal and platelet function (164). The GM can also produce neurotransmitters, such as serotonin, γ-aminobutyric acid (GABA), histamine, catecholamines, and acetylcholine, further influencing ENS activity (18). Additionally, enteric neurons express toll-like receptors (TLRs), which recognize and respond to microbial molecules (e.g., LPS and PG) or viral RNA (54).

Recent studies have employed advanced technologies to investigate how the GM regulates neural programs by sensing cues from the environment and sending this information to the CNS (165, 166). For instance, a seminal study by Muller et al. used neuronal tracing techniques to demonstrate the modulation of neuronal pathways of the MGB axis by microbes (165). Specifically, the GM was shown to influence the functioning of enteric neurons through activation of aryl hydrocarbon receptors that regulate intestinal motility (166).

The ENS can influence the HPA axis through its regulation of gut hormones, neuropeptides, and cytokines, which can stimulate or inhibit HPA axis activity (155). Conversely, the HPA axis can affect the ENS by altering gut motility and secretion, as well as modulating the activity of enteric neurons and glial cells (21). This bidirectional communication suggests that the ENS and HPA axis are closely interconnected and play important roles in regulating stress responses and maintaining homeostasis.

##### 3.2.1.2 The vagus nerve

The vagus nerve is the tenth cranial nerve that extends from its origin in the brainstem down to the visceral organs (159). It is a vital component of the PNS that connects the brain and gut to regulate homeostasis and cognitive areas function (21). The vagus nerve consists of both afferent and efferent neurons, making it the fastest and most direct pathway between the brain and gut. Evidence supports crucial roles in regulating inflammation, appetite, mood, and the stress response (167).

Some vagal endings synapse onto neurons from the ENS, and neuropod cells form fast excitatory synapses with vagal afferents using glutamate as a neurotransmitter (78, 168). This increase in the range of signals that can be transmitted by the vagus nerve enhances its ability to perform various functions. Vagal afferents express a plethora of receptors that detect various molecules such as nutrients, peptides, cytokines, hormones, and endotoxins (78). These sensory cues are transmitted rapidly to the nucleus tractus solitarius (NTS) of the brainstem (78), the primary projection site of gut-related vagal afferents in the brain (53).

Vagal brainstem nuclei then project to several regions of the brain, including nuclei involved in stress and cognition (167). For example, the hypothalamic PVN, an important hub for relaying signals from the vagus nerve, is involved in stress-induced gastrointestinal responses, including arousal, anxiety, and depression (169). Its projections to the pituitary and ventral tegmental area (VTA) provide the means to directly modulate the HPA axis and cognition, respectively. Further vagal projections to the arcuate nucleus integrate endocrine and behavioral aspects of gastrointestinal function, modulating food intake and satiety (167). Barrington’s nucleus assimilates cognitive behaviors, while the locus coeruleus maintains arousal and attention, and integrates stress and cognitive inputs. In the forebrain, the amygdala integrates emotional and aversive inputs with learning and memory. The stria terminalis processes and consolidates emotions and behavior, and regulates the HPA axis and autonomic responses to stress. The cortex integrates affect, emotion, and memory with autonomic functions (167).

The gut is a vital control center for the immune system, and the vagus nerve displays immunomodulatory properties in the complex relationship between the gut, brain, and inflammation (170). In response to cytokines and endotoxins, the vagus nerve signals the brainstem, affecting fever and sickness behavior, as well as appetite and mood (171–174). The HPA axis reacts by providing a modulatory anti-inflammatory response (175–177). Moreover, the cholinergic anti-inflammatory pathway primarily signals through vagal efferents, serving as the effector limb of the “inflammatory reflex” and interfacing the nervous and immune systems (50, 170, 178–182) (**Figure 1**).

Animal studies have shown that disrupting the vagus nerve can lead to abnormalities in neurogenesis, stress reactivity, cognition, and anxiety- and fear-related behavior (183). On the other hand, stimulating the vagus nerve has been found to enhance memory (184, 185), facilitate hippocampal neurogenesis, increases expression of brain-derived neurotrophic factor (BDNF) (186, 187), and enhance synaptic plasticity (188). BDNF is an important plasticity-related protein that promotes neuronal growth, development, and survival, and plays a key role in learning, memory and mood regulation. Altered BDNF expression is associated with disruptions in cognitive function (189). These findings suggest that the vagus nerve promotes neurogenic and neurotrophic signaling. Indeed, vagal nerve stimulation is used to treat refractory epilepsy, Crohn’s disease, refractory depression, chronic pain, and other conditions in humans (159).

This section highlights the role of the GM in regulating brain function through the vagus nerve. Animal and human studies have demonstrated that the vagus nerve serves as the primary and most direct signaling pathway between the GM and the brain (60, 78, 115, 190). The GM can activate vagal afferents directly or indirectly by releasing neuroactive mediators or by influencing the luminal concentration of molecules that vagal afferents detect (28, 78). Specific bacterial strains have been shown to influence vagus nerve signaling, to communicate with the brain, and alter cognition. For example, administration of *Citrobacter rodentium*, a pathogen, increased anxiety-like behaviors in mice, while *Bifidobacterium longum* (NC3001) produced anxiolytic effects in a vagus-dependent manner (191, 192). Similarly, *Campylobacter jejuni* administration resulted in increased levels of anxiety-related behavior and activation of vagal afferents (178). Additionally, studies have shown that vagotomy prevented the positive effects of administration of a human milk oligosaccharide on long-term potentiation, learning, and memory in rats (193).

#### 3.2.2 Neurotransmitters

**Figure 2:**
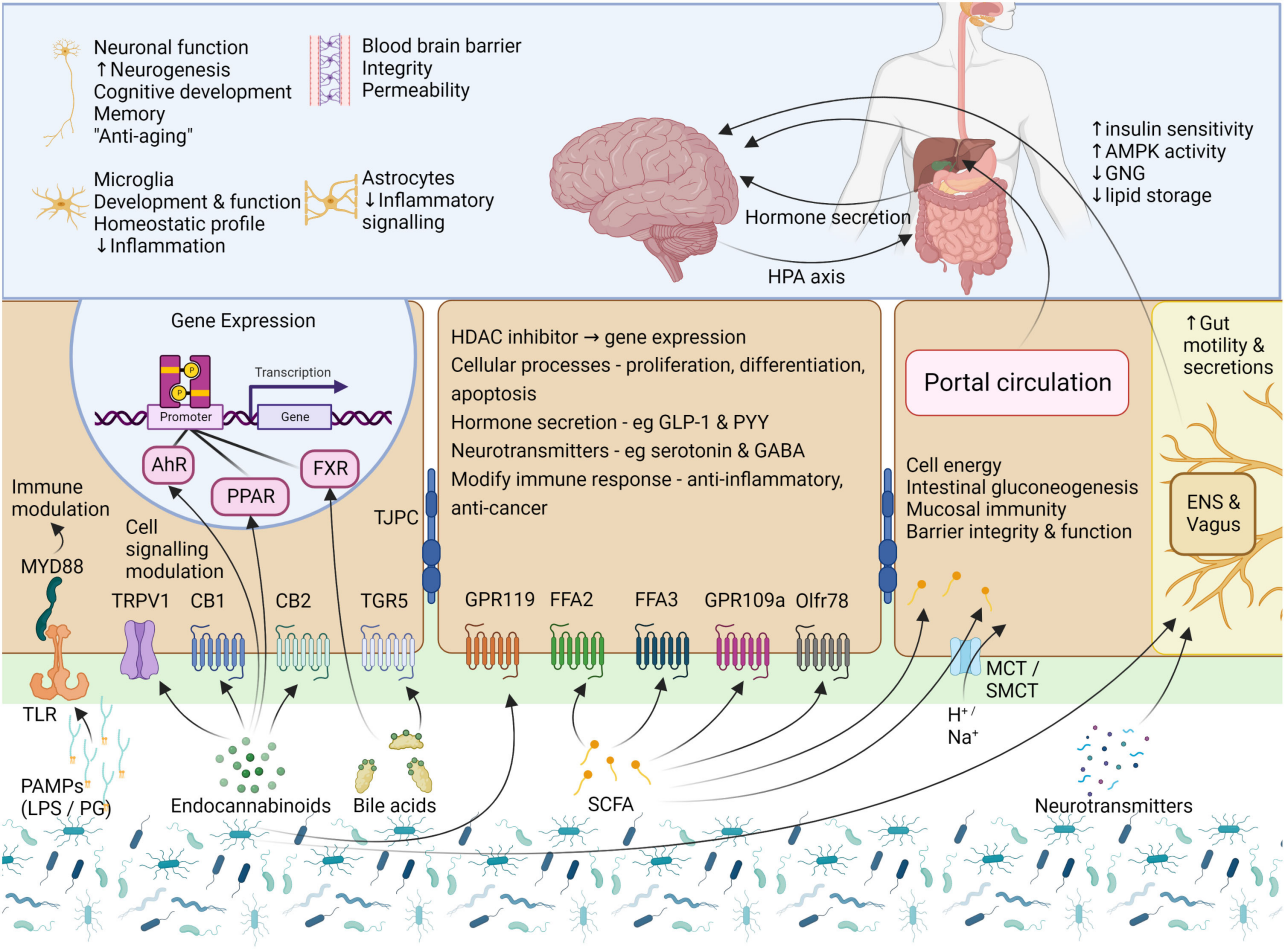
Signalling mechanisms – microbial products, metabolites, and neurotransmitters. Cells of the gut express a variety of receptors which are able to sense and transmit signals from the intestinal lumen and mucosa. To communicate, the GM uses factors which include several microbial products, eCBs, BAs, SCFAs, and neurotransmitters. PAMPs, such as LPS and PG, are small molecular microbial motifs that are recognized by TLRs, while this signal is transferred to intracellular signaling pathways (for e.g., immune cell activation) by MYD88. The eCB system is not limited to the activity of CB1 and CB2, and eCBs can also interact with other GPCRs, TRPV1, and the nuclear receptors PPAR-α and PPAR-γ. To modulate gut function, BAs interact with two main receptors, the GPCR named TGR5, and the nuclear receptor FXR. In the gut, SCFAs can activate FFA2, FFA3, GPR109a and Olfr78, but may also enter the cell *via* transporters or *via* passive diffusion where they modulate the activity of several enzymes and transcription factors or provide a source of energy for the cell. Small amounts of SCFAs are taken up into circulation where they may be transported to target tissues such as the liver, pancreas and brain. The binding of these GM-derived molecules with their respective receptors leads to the activation of cellular signaling pathways which then leads to alterations in cellular activity and gene expression, with downstream effects on host physiological processes. AhR, aryl hydrocarbon receptor; AMPK, AMP-activated protein kinase; BA, bile acid; CB1 and CB2,, cannabinoid receptor type 1 and 2; eCB, endocannabinoid; ENS, enteric nervous system; FFA2 and FFA3, free fatty acid receptor 2 and 3; FXR, farsenoid X receptor; GABA, γ-aminobutyric acid; GLP-1, glucagon-like peptide 1; GNG, gluconeogenesis; GPR119 and GPR109a, G-protein coupled receptor 119 and 109a; HDAC, histone deacetylase; LPS, lipopolysaccharide; MCT, monocarboxylate transporter; MYD88, Myeloid differentiation primary response 88; Olfr78, Olfactory receptor 78; PAMP, Pathogen-associated molecular pattern; PG, peptidoglycan; PPARα/γ, peroxisome proliferator-activated receptors α/γ; PRRs, pattern recognition receptors; PYY, Peptide YY; SCFA, short chain fatty acid; SMCT, sodium-dependent monocarboxylate transporter; TGR5, Takeda G protein-coupled receptor 5; TJPC, tight junction protein complex; TLR, toll-like receptor; TPRV1, transient receptor potential cation channel subfamily V member 1. Figure created with BioRender.com.

Neurotransmitters provide additional communication mechanisms between the GM and nervous system (**Figure 2**). Microbes synthesize and metabolize several neurotransmitters, including dopamine, noradrenaline, serotonin, acetylcholine, histamine, and GABA (28). However, these neurotransmitters do not seem to cross the BBB and likely act indirectly to modulate brain function *via* the vagus nerve or ENS (78). Some neurotransmitter precursors synthesized in the gut may reach the CNS *via* the circulation and are able to cross the BBB *via* active transporters (194).

Animal studies provide evidence that microbial modulation of these neurotransmitters may impact host physiology, and preliminary human studies demonstrate that microbiota-based interventions can alter neurotransmitter concentrations (28). Germ-free mice studies have shown significant alterations in multiple neurotransmitter systems and their receptors in several brain regions (124). Similarly, antibiotic administration to deplete the GM can change the levels of neurotransmitters in the gut and blood (195, 196). Furthermore, microbial abundance has been shown to alter the expression of neurotransmitter receptors in the brain (51, 189, 191). Therefore, there is a growing body of evidence suggesting that the GM can ultimately influence the levels of neurotransmitters in the brain and alter brain function and cognition.

##### 3.2.2.1 Tryptophan metabolism

Tryptophan is an essential amino acid. Its synthesis by microbes has been well described (197). In the gut, it may be further metabolized under direct or indirect control by the GM, giving rise to several compounds, such as serotonin, kynurenines, tryptamine, and indolic compounds, which participate in MGB communication (197, 198).

**Figure 3:**
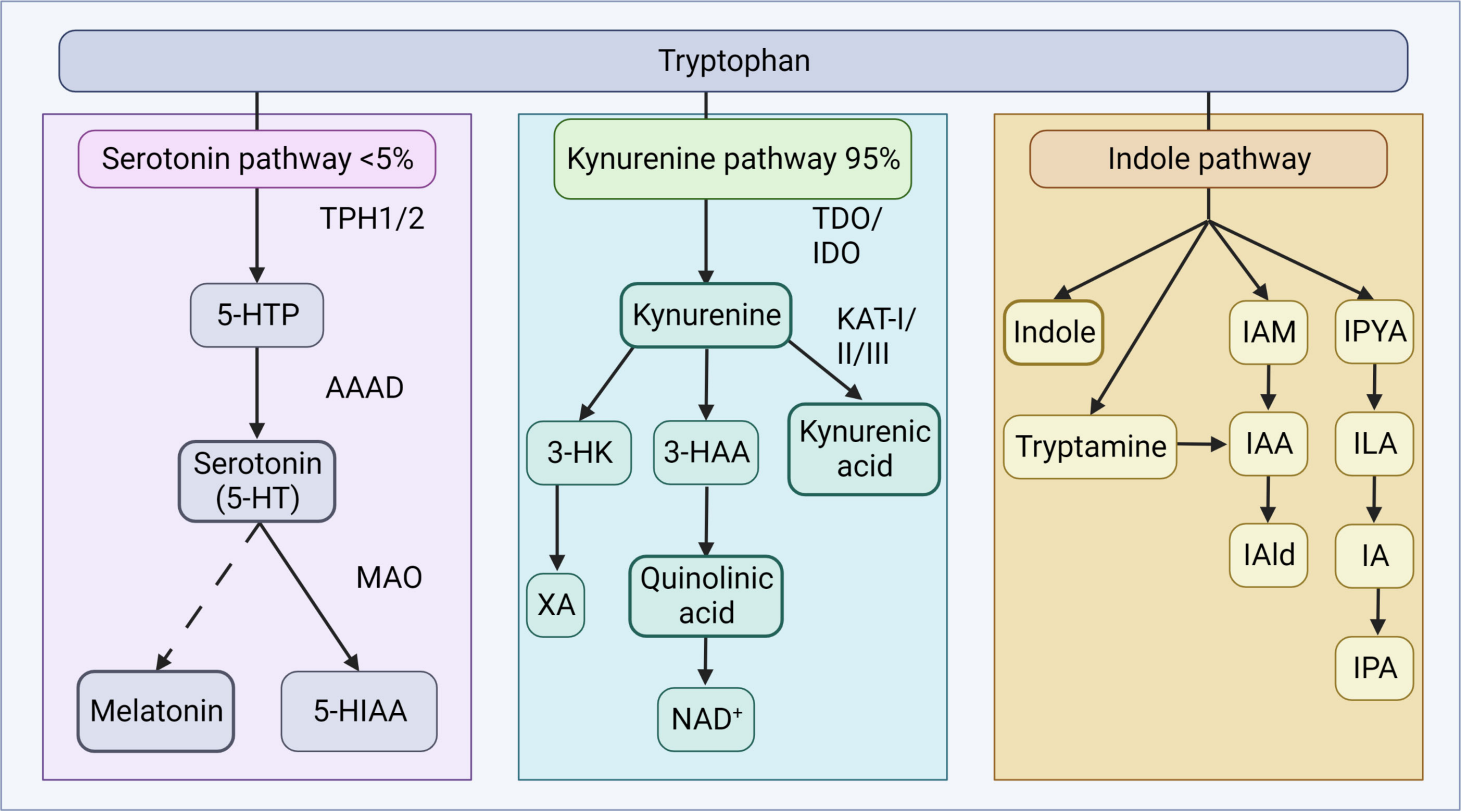
Tryptophan metabolism. Tryptophan metabolism occurs *via* the serotonin or kynurenine pathways to produce bioactive products. In the serotonin pathway, tryptophan is converted to 5-HTP by TPH1 in enterochromaffin cells, or TPH2 in neurons of the ENS or CNS. AAAD converts 5-HTP to serotonin, which can be further metabolized to melatonin, *via* a series of steps. The vast majority of tryptophan is, in fact, utilized in the kynurenine pathway, where tryptophan is converted to kynurenine by TDO in the liver (majority), or ubiquitously *via* IDO (including gut, brain, liver). Kynurenine can be converted to kynurenic acid by the KAT enzymes, quinolic acid and further NAD^+^, or XA. In the indole pathway, microbes of the gut metabolize tryptophan into indole and indole derivatives. 3-HAA, 3-hydroxyanthranilic acid; 3-HK, 3-hydroxykynurenine; 5-HIAA, 5-hydroxyindoleacetic acid; 5-HT, 5-hydroxytryptamine; 5-HTP, 5-hydroxytryptophan; AAAD, aromatic amino acid decarboxylase; IA, anholocyclic acid; IAA, indole-3-acetic acid; IAAld, indole-3-acetaldehyde; IAld, indole-3-aldehyde; IAM, indole-3-acetamide; IDO, indoleamine 2,3-dioxygenase; ILA, indole-3-lactic acid; IPA, indole-3-propionic acid; IPYA, indole-3-pyurvic acid; KAT, kynurenine aminotransferase; MAO, monoamine oxidase; NAD^+^, nicotinamide adenine dinucleotide; TDO, tryptophan 2,3-dioxygenase; XA, xanthurenic acid. Figure created with BioRender.com.

Although tryptophan is essential for serotonin synthesis, the dominant physiological pathway is the kynurenine pathway (**Figure 3**). Kynurenine is produced from tryptophan by the action of the hepatic enzyme, tryptophan-2,3-dioxygenase (TDO), or the ubiquitous indoleamine-2,3-dioxygenase (IDO) (199). Glucocorticoids and tryptophan induce TDO, while cytokines induce IDO (200). Kynurenine can cross the BBB, and is further metabolized along two separate arms to either kynurenic acid or quinolinic acid, and further, niacin and nicotinamide adenine dinucleotide (NAD^+^) (197). The balance between these two metabolites appears important in neural health and disease (31), as kynurenine pathway end-products are implicated in the regulation of biological processes involving neurotransmission, inflammation, and immunity (197). Moreover, kynurenic acid appears to exert mucosal protective and immunoregulatory effects in the gut (201). Activation of stress-responsive TDO or immune-responsive IDO can limit the availability of tryptophan for serotonin synthesis and increase the downstream production of neurotoxic or neuroprotective metabolites (31).

In a recent study, the importance of the GM’s ability to metabolize tryptophan into aryl hydrocarbon receptor ligands and, therefore, modulate gut inflammation, was demonstrated in patients with celiac disease (202). Several bacterial taxa can also affect tryptophan levels by direct utilization for growth or *via* tryptophanase expression (28, 203, 204), and these bacteria have been associated with the development of neuropsychiatric disorders, including autism spectrum disorders (205). Mounting evidence suggests that the GM modulates the tryptophan and kynurenine pathways and is a humoral route through which the GM may influence cognition at the level of the CNS (206–210).

##### 3.2.2.2 Serotonin

Serotonin regulates pleiotropic physiological processes, including cognition, circadian rhythm, nociception, blood coagulation, cardiovascular homeostasis, and gastrointestinal secretion and peristalsis (211). Serotonin effects are mediated by the family of serotonin G-protein-coupled receptors (GPCRs) (212). Serotonin exerts both central and peripheral control. The vast majority of serotonin is found outside of the CNS, with 90-95% located in the gut, mostly within enterochromaffin cells (213, 214).

Serotonergic neurons have a significant influence on neuroendocrine function, and there is a dynamic interplay and extensive crosstalk between the serotonergic system and HPA axis (215). Serotonin has complex effects on the overall stress response, depending on the target cell and receptor type (216). Serotonin regulates upstream CRH signaling systems *via* the activation of serotonin 2C receptors of the hypothalamic PVN (217). Pre-gestational and early-life stress, with activation of the HPA axis, have been linked with an altered serotonergic system, leading to interruptions in brain development and cognition (218–220). Indeed, the development of the serotonergic system within the MGB axis depends on a low-stress environment and early life events may be critical.

The GM can further regulate serotonin availability by signaling enterochromaffin cells to produce serotonin *via* expression of tryptophan hydroxylase, and by altering levels of SCFAs and BAs which can influence serotonin production (46, 164, 221–223). Germ-free mice exhibit reduced colonic serotonin production and decreased levels in the blood, which normalize with microbial colonization (164, 223). Moreover, male germ-free mice have increased hippocampal serotonin levels, which colonization immediately post-weaning does not reverse (124). When administered to rats, *B. infantis* results in reduced 5-HIAA (serotonin metabolite) concentrations in the frontal cortex, and a marked increase in blood concentrations of tryptophan and kynurenic acid (224). Thus, the GM can indirectly influence the activity of the serotonergic system, which in turn can affect the HPA axis and stress response.

##### 3.2.2.3 Catecholamines

Catecholamines, such as dopamine and noradrenaline, regulate various body functions, including cognition, mood, and gut motility and integrity (225). Dopamine is a major neurotransmitter in reward-motivated behavior and is a precursor for other catecholamines, like noradrenaline and adrenaline. Noradrenaline is involved in arousal, alertness, sensory signal detection, behavior, cognition, and the acute stress response (226). It is well established that brainstem catecholaminergic centers play an important role in the regulation of the HPA axis (227) and noradrenergic neurons are required for normal activation in response to a variety of stressors. Noradrenaline, released within the intestinal wall during activation of the sympathetic nervous system in acute stress, has a wide variety of actions at the intestinal mucosa, such as modulating intestinal motility and transepithelial ion transport (228).

The GM also synthesize and respond to catecholamines. For example, bacteria produce noradrenaline as a quorum sensing molecule, and noradrenaline and adrenaline can promote pathogenesis and growth (229, 230) Germ-free mice show decreased noradrenaline in the cecal lumen and tissue, which is restored with colonization (231). Additionally, they have increased turnover rates of dopamine, noradrenaline, and serotonin in the brain (124).

##### 3.2.2.4 γ-aminobutyric acid

γ-aminobutyric acid (GABA) is an inhibitory neurotransmitter synthesized from glutamate by GABAergic neurons in the brain. GABA regulates various physiological processes and has been shown to play a central role in cognition by regulating and synchronizing neuronal signaling in the hippocampus (232). The HPA axis is also regulated by GABAergic signaling at the level of CRH, and nearly 50% of all synapses in the PVN are GABAergic (233). Although CRH neurons integrate information from many different brain regions involving several neurotransmitter systems, the activity of CRH neurons is ultimately regulated by GABAergic inhibition, mediated by GABA_A_ receptors (234). The HPA axis can also influence GABA production and signaling. Altered GABAergic profiles are associated with multiple diseases with cognitive dysfunction, such as dementia and depression.

The GM can metabolize GABA and recent research has shown that certain microbes can produce GABA, required for their growth (235). In addition, changes in GM composition have been associated with alterations in GABA receptor expression and GABA levels in the brain (191). Germ-free animals demonstrate decreased GABA concentrations in stool and blood, while fecal GABA levels can be modified with antibiotics. Remarkably, in a study of healthy women, levels of *Bacteroides*, identified as the major microbial producers of GABA, were associated with increased grey matter in the cerebellum, hippocampus, and frontal regions of the brain, as well as reduced levels of anxiety, distress, and irritability (236). GABA does not cross the BBB and so microbial-derived GABA would need to act locally on the ENS or vagus nerve to influence the CNS. However, SCFAs such as acetate can cross the BBB and be incorporated into the GABA metabolic cycle (237).

#### 3.2.3 The endocannabinoid system

**Figure 4:**
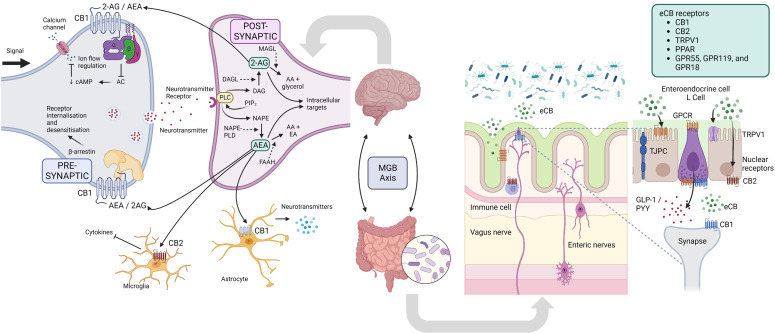
Endocannabinoid system. In the nervous system, presynaptic electrical impulses lead to calcium entry into the cell which drives the release of neurotransmitters into the synapse. Neurotransmitter receptors on the postsynaptic neurons are then activated and drive the action potential forward. The eCB system is a ubiquitous neuromodulatory system that functions throughout the body, including the nervous system to modulate cell signaling. DAG and NAPE are produced from phospholipid precursors, and are converted to the endocannabinoids (eCB) 2-AE and AEA by DAGL and NAPE-PLD, respectively. In retrograde signaling, these eCBs are mobilized from postsynaptic neurons and target presynaptic CB1 receptors to suppress neurotransmitter release by inhibiting AC, decreasing cAMP and therefore decreasing calcium ion flow into the cell, or alternatively influence receptor sensitivity and internalization *via* β-arrestin. eCB signaling in the CNS can also affect the functioning of microglia and astrocytes, with modulation of the release of cytokines and neurotransmitters, respectively. In the gut, eCBs secreted by certain microbes (or host cells) interact in microbiota-epithelial crosstalk, and include the immune and nervous systems, and metabolic, endocrine and barrier functions. 2-AG, 2-Arachidonoylglycerol; AA, arachidonic acid; AEA, N-arachidonoylethanolamine (aka anandamide); AC, adenylate cyclase; CNS, central nervous system; cAMP, cyclic AMP; DAG, diacylglycerol; DAGL, diacylglycerol lipase; EA, ethanolamine; FAAH, Fatty acid amide hydrolase; GLP-1, glucagon-like peptide 1; GPCR MAGL - monoacylglycerol lipase; MGB, microbiota-gut-brain; NAPE, N-Acyl-phosphatidylethanolamine; NAPE-PLD, NAPE phospholipase D; PIP2, Phosphatidylinositol 4,5-bisphosphate; PLC, phospholipase C; PYY, Peptide YY; TPRV1, transient receptor potential cation channel subfamily V member 1. Figure created with BioRender.com.

The endocannabinoid system (ECS) is a complex signaling system found throughout the body. The ECS is composed of endocannabinoids (eCBs), cannabinoid receptors, and enzymes involved in the synthesis and degradation of endocannabinoids. The two primary endocannabinoids are anandamide (AEA) and 2-arachidonoylglycerol (2-AG) (238). These bioactive lipid mediators are produced from the common phospholipid precursor arachidonic acid and released by various cell types in the body, including neurons, immune cells, and adipocytes (239). They bind high-affinity GPCRs, including cannabinoid receptors type 1 (CB1) and type 2 (CB2) (238). As neuromodulators, eCBs often act in retrograde, released from postsynaptic cells and traveling backward across synapses, where they transiently inhibit the release of either inhibitory GABA or excitatory glutamate from presynaptic terminals (240) (**Figure 4**).

The ECS modulates a multitude of physiological processes, including the HPA axis (241), cognition, learning and memory (242), intestinal-barrier function (243), inflammation (244), energy metabolism (245), among others (reviewed recently (239)). In response to stress, eCB signaling modulates glucocorticoid and CRH signaling in the brain and is crucial in recovering homeostasis (241, 246, 247). The ECS is also widely expressed in neural tissue of the gut and is critically involved in the maintenance of intestinal homeostasis. It regulates barrier function and permeability through the immune system, epithelial tight junction proteins, and mucous secretion (248). Furthermore, it modulates myenteric neuron activity, SNS and vagal nerve function, and the release of neuropeptides such as ghrelin, leptin, and orexin (249).

The ECS and GM interact to regulate intestinal homeostasis resulting in relevant functional effects in the gut and CNS (238, 246, 248, 250). Dysbiosis affects eCB signaling, and vice versa (243). Germ-free animals demonstrate significant changes in the expression of CB1 and CB2, and synthetic and degradative enzymes throughout the gut (251). Vijay et al. studied the relationships between the ECS, inflammatory cytokines, and the GM using a six-week exercise intervention in humans (252). Changes in eCBs correlated with increased butyrate levels, and decreased TNF, IL-6 and IL-10. Hence, the anti-inflammatory effects of SCFAs may be partly mediated by the ECS. Healthy mice colonized with *Candida albicans* showed marked anxiety-like behavior and increased corticosterone concentrations that were inversely correlated with forebrain AEA, demonstrating disruption of the HPA axis through dysregulation of the ECS (253). Both animal and human studies have shown that the ECS and GM play a role in cognitive decline (254). Although microbes secrete eCBs, their role in host physiology remains unclear (255).

### 3.3 The immune system

The immune system’s primary responsibility is to distinguish between “harmful” and “harmless” signals and respond appropriately. This is especially important in the gut, where immune cells are constantly in contact with microbes. The GM is therefore closely linked to the immune system, and they interact in several ways [reviewed recently (256, 257)]. Additionally, the immune system, HPA axis and CNS, and GM are closely interlinked.

One critical interaction involves the activation of pattern recognition receptors (PRRs), including TLRs, by microbial associated molecular patterns (MAMPs) (54). These molecular signatures consist of microbial products such as the endotoxins LPS and polysaccharide A (on the cell surface of gram-negative bacteria) and PG (on gram-positive bacteria) (258). Each MAMP is detected by a specific PRR expressed on various cell types, including cells of the immune system (macrophages and natural killer cells) and nervous system (myenteric neurons and enteric glial cells) (53).

Cytokines and chemokines are another mechanism by which the immune system, GM, and CNS (including HPA axis) interact (259). Immune cells in the gut produce cytokines to maintain intestinal homeostasis, which in turn affects local microbial concentrations (260). Cytokines may also be produced locally in the CNS, or they may cross the BBB from the systemic circulation, and directly affect brain function.

Epithelial integrity is a crucial feature of gastrointestinal and nervous system homeostasis. It is essential to prevent the unregulated leakage of products across the barrier while allowing the transport of essential molecules. Furthermore, gut epithelial integrity is critical for maintaining the symbiotic relationship with the commensal microbes of the GM. This physical barrier includes the mucosa, epithelial cells, as well as tight junction proteins, such as occludin, claudins and zonula occludens (261). These tight junction protein complexes are dynamically modulated by intracellular signaling transduction systems and several extracellular stimuli, including cytokines, small GTPases, and post-translational modifications. When these regulatory mechanisms break down, barrier integrity may be compromised. Injury, infections and autoimmune diseases can influence the permeability of the gut and BBB (262). Microbes and microbial products then gain access to the circulation and may gain easier access to the CNS (262). Moreover, microbial products, cytokines, and other immune molecules released under the influence of the GM may further influence the BBB’s integrity, alter BBB transport rates, and promote the release of neuroimmune molecules from the cells of the BBB (262). The GM can also alter BBB permeability by changing the expression of occludin and claudin 5 (263). These factors may lead to neuroinflammation, which is an important process shaping brain function.

Crosstalk between the GM and CNS is also essential for normal development and homeostatic functioning of the immune system, both innate and adaptive (256). While immune cell activation and cytokine production have a minor impact on the CNS during physiological perturbations, chronic systemic inflammation, mainly in the form of infections, has long been associated with behavioral alterations and cognitive dysfunction (264–266). Antibiotic-treated and germ-free mice have pro-inflammatory systemic and CNS immune system responses (120, 267). Perturbations in microbial diversity, secondary to antimicrobials, have been shown to influence pro-inflammatory cytokine secretion in the CNS and alter microglial morphology (268–270).

### 3.4 Microbial metabolites

In addition to the complex communication pathways between the GM and the host immune and nervous systems that have been described, there are several other mechanisms involving the production of small molecules that impact human function (271, 272).

#### 3.4.1 Short chain fatty acids

Short chain fatty acids (SCFAs) are small organic monocarboxylic acids produced by bacterial fermentation of non-digestible polysaccharides in the large intestine. The main SCFAs are butyrate (C4), propionate (C3), and acetate (C2) (132). SCFAs are absorbed by colonocytes *via* monocarboxylate transporters (MCTs) or *via* non-ionic diffusion across the epithelium (131, 273).

SCFAs are a source of energy and trophic factors for cells of the colon and liver (274). Additionally, they can bind GPCRs, specifically the free-fatty-acid receptors FFA2, FFA3, Olfr78, and GPR109a, located throughout the body, including enteroendocrine, immune, and neural cells (275–278). This suggests that SCFAs play a key role in neuro-immuno-endocrine regulation (279–282) (**Figure 4**). Indeed, extensive evidence supports pleiotropic roles of SCFAs, which affect several host organs and systems, including the gut and CNS (127, 131, 132). SCFAs have several local effects that improve intestinal health, including the maintenance of intestinal barrier integrity, mucus production, and protection against inflammation (250). These processes are crucial to the gut’s first line of defense. SCFAs promote immunity and suppress inflammatory responses in the intestine and other organs by regulating immune cells such as lymphoid cells, T cells, and B cells (283–285).

By inhibiting histone deacetylase (HDAC) activity, SCFAs also regulate systemic functions, promoting histone acetylation and gene expression in host cells (250). This epigenetic mechanism has been described in gastrointestinal, immune and neurological cells [reviewed (131)].

SCFAs appear to play a significant role in MGB communication (286). Research indicates that SCFAs can indirectly modulate the PNS through expression of FFA3 in the enteric neural plexus, portal nerve, and autonomic and sensory ganglia (131). Activation of FFA3 receptors on vagus nerve cells can result in the activation of various neurons in the CNS, including dynamic regulation of hypothalamic neuronal circuitry (287).

SCFA-induced activation of receptors on enteroendocrine cells can promote gut-brain signaling by inducing hormones such as glucagon-like peptide 1 (GLP1) and peptide YY (PYY), as well as neurotransmitters like GABA and serotonin (276). SCFA-signaling can also induce other hormones, including leptin from adipocytes, and insulin from pancreatic β-cells (288). Additionally, SCFAs can modulate the levels of neurotransmitters and neurotrophic factors and regulate the expression of tryptophan 5-hydroxylase, the enzyme involved in the synthesis of serotonin, and tyrosine hydroxylase, the enzyme involved in the rate-limiting step in dopamine, noradrenaline, and adrenaline synthesis (18, 132, 164, 222).

Moreover, the abundant expression of MCTs on endothelial cells suggests that SCFAs can cross the BBB, which is supported by the presence of SCFAs in human cerebrospinal fluid (CSF) and in brain uptake studies (286). Accumulating evidence supports the idea that SCFAs are necessary for the maintenance of CNS homeostasis, learning and cognition, and reward-associated behaviors (25). SCFAs also influence the integrity of the BBB by upregulating the expression of tight junction proteins (263).

SCFAs may also improve neuronal homeostasis and function by influencing neurotrophic factors such as nerve growth factor, glial cell line-derived neurotrophic factor, and BDNF (119–122). These factors regulate the growth, survival, and differentiation of neurons and synapses in the CNS, and are important for learning and memory. SCFAs can modify neuroinflammation by affecting the morphology and function of glial cells (120, 286, 289, 290). By administering SCFAs to germ-free mice, Erny et al. were able to rescue deficits in microglial immaturity and morphology (120).

There is also evidence to suggest that SCFAs can modulate the HPA axis. In stressed mice, SCFA administration reduced HPA axis hyperactivity and intestinal permeability (291). In humans, a recent triple-blind, randomized, placebo-controlled intervention trial examined the effects of colonic SCFA-mixture delivery in men on responses to psychosocial stress and fear tasks (292). SCFA supplementation was shown to downregulate the HPA axis by significantly attenuating the cortisol response.

Altered SCFA production has also been demonstrated in a variety of neuropathologies (42, 116, 127, 130, 132, 292–295). These findings suggest that SCFAs regulate CNS processes through both direct and indirect mechanisms and may ultimately affect host cognition and response to stress.

#### 3.4.2 Bile acids

**Figure 5:**
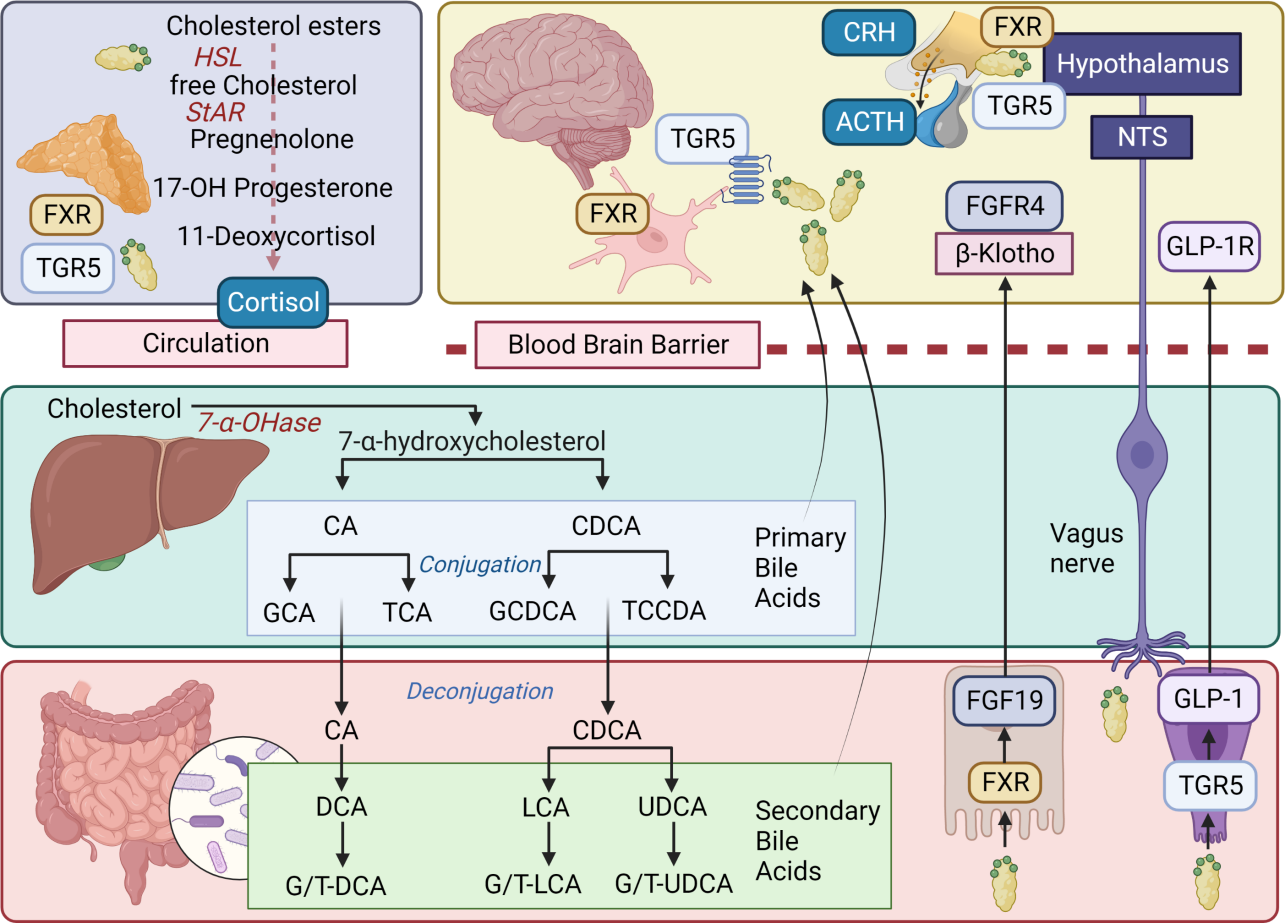
Bile acids, BA receptors, and signaling pathways. In the liver, the classical pathway of bile acid (BA) synthesis begins with the conversion of cholesterol into 7α-hydroxycholesterol by the rate-limiting enzyme cholesterol 7α-hydroxylase (7α-OHase; CYP7A1). The 7α-hydroxycholesterol is then further metabolized into cholic acid (CA) and chenodeoxycholic acid (CDCA) through a series of enzymatic reactions. Once synthesized, BAs are conjugated with either glycine or taurine, which increases their solubility and reduces their toxicity. The conjugated BAs are then secreted into bile canaliculi, stored in the gallbladder, and released into the small intestine following a meal. After completing their role, approximately 95% of BAs are reabsorbed in the ileum and transported back to the liver *via* the enterohepatic circulation. As BAs pass through the gastrointestinal tract, they encounter a diverse population of gut bacteria and the synthesis of secondary BAs occurs in the large intestine as a result of microbial biotransformation. Secondary BAs are important for maintaining the overall BA pool in the body and contribute to the regulation of cholesterol homeostasis, energy metabolism, and the immune system. BAs can also act as signaling molecules, interacting with specific receptors such as the nuclear receptor FXR and the cell membrane receptor TGR5 (expressed in various tissues, including the liver, gut, enteric nervous system, CNS, and adrenal glands) which are involved in the modulation of numerous physiological processes, including glucose metabolism, lipid metabolism, and the regulation of the gut-brain axis. In the gastrointestinal tract, BAs bind FXR in enterocytes and this activates the expression of FGF19, which is then secreted into the bloodstream and plays a crucial role in MGB communication. FGF19 acts as an endocrine signal crossing the BBB to reach the CNS and then binding to its cognate receptor, FGFR4, and co-receptor β-Klotho. This interaction leads to the activation of intracellular signaling cascades, such as the MAPK pathway and the PI3K/Akt pathway. These signaling pathways regulate various processes, including cell growth, differentiation, and metabolism, and contribute to the modulation of the gut-brain axis. Additionally, activation of TGR5 by BAs can lead to the release of GLP-1, an incretin hormone that modulates insulin secretion and glucose homeostasis. In the CNS, TGR5 activation has been implicated in the regulation of energy balance, neuroinflammation, and neuroprotection. BAs can influence the HPA axis through both direct and indirect mechanisms involving signaling pathways in the CNS and the adrenal glands. In the CNS, BAs can modulate the HPA axis by interacting with FXR and TGR5, which are expressed in various brain regions, including the hypothalamus and the hippocampus. Activation of these receptors by BAs can influence the release of CRH from the hypothalamus and ACTH from the pituitary gland, leading to the modulation of cortisol secretion from the adrenal cortex. Furthermore, BAs can directly affect the adrenal glands, influencing the release of cortisol. BA can alter adrenal steroidogenesis by modulating the expression and activity of key enzymes involved in the biosynthesis of cortisol, including HSL, StAR, and cytochrome P450 enzymes (e.g., CYP11A1, CYP11B1, and CYP11B2). Additionally, BAs can influence adrenal cell function by activating FXR and TGR5, which may regulate intracellular signaling pathways and gene expression patterns related to steroid hormone production, inflammation, and oxidative stress. Primary bile acids: CA, cholic acid; CDCA, chenodeoxycholic acid; GCA, glycocholic acid; TCA, taurocholic acid; GCDCA, glycochonedeoxycholic acid; TCCDA, taurochenodeoxycholic acid. Secondary bile acids: DCA, deoxycholic acid; G/T-DCA, glyco/tauro-deoxycholic acid; G/T-LCA, glyco/tauro-lithocholic acid; G/T-UDCA, glyco/tauro-ursodeoxycholic acid; UDCA, ursodeoxycholic acid; LCA, lithocholic acid; UDCA, ursodeoxycholic acid. ACTH, adrenocorticotropic hormone; Akt, protein kinase B; BA, bile acid; BBB, blood brain barrier; CNS, central nervous system; CRH, corticotrophin-releasing hormone; FGF19, fibroblast growth factor 19; FGFR1-4, fibroblast growth factor receptors 1 to 4; FXR, farnesoid X receptor; GLP-1, glucagon-like peptide 1; GLP-1R, glucagon-like peptide 1 receptor; HSL, hormone sensitive lipase; MAPK, mitogen-activated protein kinase; MGB, microbiota-gut-brain; PI3K, phosphatidylinositol 3-kinase; StAR, steroidogenic acute regulatory protein; TGR5, Takeda G protein-coupled receptor 5. Figure created with BioRender.com.

Bile acids (BAs) are products of cholesterol metabolism primarily produced in the liver as primary BAs and modified by the GM into secondary BAs through processes such as deconjugation, dihydroxylation, dehydrogenation, and isomerization (**Figure 5**) (30, 296, 297). While their role in enterohepatic circulation as detergents for lipid digestion is well established, recent studies have also revealed their function as hormones *via* receptors such as farnesoid X receptor (FXR) and Takeda G protein-coupled receptor 5 (TGR5), with significant regulatory and signaling activities (298). BAs can also activate pregnane X receptors, vitamin D receptors, and glucocorticoid receptors (299). Their functions encompass regulation of motor, sensory, and secretory functions of the gut, intestinal barrier permeability, inflammatory response, and several metabolic processes, including lipid and glucose metabolism, and hepatic gluconeogenesis (300).

The effects of BAs extend beyond the gut, impacting various tissues throughout the host. BA receptors are present in the brain, and BAs can either be synthesized locally or actively transported across the BBB by BA transporters from circulation (297, 301, 302). Consequently, circulating BA levels significantly influence the CNS’s BA profile (303). FXR knockout mice exhibit abnormal BA and neurotransmitter concentrations, resulting in impaired cognition and motor coordination (304). TGR5, expressed in brain and peripheral neurons, as well as glial and microglial cells, can be activated by several neurosteroids (305). Specific BAs demonstrate neuroprotective effects in cellular and animal models, with human clinical trials underway (306–308).

BAs play a role in regulating the HPA axis. BAs modulate HPA axis activity by inhibiting CRH release through FXR activation, expressed in the hypothalamus (309). Additionally, BAs can interact with TGR5, expressed in the hypothalamic PVN, stimulating the HPA axis by increasing CRH (310, 311). Cholestasis, associated with suppression of the HPA axis, likely due to BA interactions with glucocorticoid receptors in the brain (299, 312). The discovery of FXR and TGR5 receptors in the adrenal gland further connects BAs with glucocorticoid metabolism (311, 313–315). BAs might act *via* TGR5 in a cAMP/protein kinase A (PKA)-dependent fashion phosphorylating and thus activating steroidogenic acute regulatory protein (StAR) and hormone sensitive lipase (HSL) (316). FXR activation is known to regulate lipoprotein receptors and transporters, as well as enzymes in the steroidogenic pathway, and has been shown to increase corticosterone levels in mice (311).

The GM influences BA metabolism, and BAs affect the GM’s composition (296). Specific microbes directly contribute to BA transformation, impacting the BA pool’s composition and size (317). Some BAs serve as substrates for gut microbes, while others exhibit antimicrobial properties, actively shaping the GM at the highest taxonomic levels (318). The BA-microbiota axis modulates the immunoregulatory environment along the gut (303). Given this close bidirectional relationship between BAs and the GM, these metabolites have emerged as important modulators of the MGB axis, functioning directly *via* BA receptors in the ENS and brain or indirectly *via* GLP-1 or the FXR-FGF15/19 axis (297, 319). Changes in the GM’s composition correspond to changes in blood and brain BA profiles, which are essential because specific BA ligands’ distinct physicochemical properties determine the potency of BA receptor activation (320–323).

Altered BA profiles have been observed in several neuropathologies associated with cognitive decline, such as Alzheimer’s disease (302, 324). BA disorders are also associated with neural symptoms (300). BAs may substantially affect cognitive function by their affinity to muscarinic receptors, as well as GABA and NMDA receptors (325). Germ-free mice excrete less fecal BAs, have a larger BA pool, and have different gene expression profiles involved in BA metabolism, than wild-type mice (323). Postnatal maturation of the GM in newborn mice was shown to be dependent on BAs and neonatal cholestasis is associated with dysbiosis in infants (326, 327). Bile duct ligation alters the GM composition, and increases the permeability of the BBB (328, 329). In a study of patients with depression and anxiety, BA profiles associated with altered GM composition were significantly different in those with more severe symptoms, and specific BA parameters were able to distinguish treatment failures from remitters (330).

#### 3.4.3 Branched-chain amino acids

Branched-chain amino acids (BCAAs) are essential amino acids including leucine, isoleucine, and valine, which participate in various biochemical functions, including energy production, protein synthesis, insulin secretion, brain amino acid uptake, and immunity (331). The GM produces higher proportions of specific BCAAs (valerate, isobutyrate, and isovalerate) relative to other amino acids, which have been shown to influence epithelial and mucosal homeostasis (332). Additionally, BCAAs can be utilized by microbes, potentially regulating intestinal microbial species, diversity, and metabolism (333, 334).

BCAAs regulate key signaling pathways, most notably the activation of mechanistic target of rapamycin (mTOR), which serves as the master regulator of cell growth and proliferation (331). Mice supplemented with a BCAA-enriched cocktail exhibited improved physical endurance and an extended lifespan (335).

In the CNS, BCAAs play roles in protein synthesis, food intake regulation, and serve as nitrogen donors involved in intercellular shuttling and the synthesis of the neurotransmitters glutamate and GABA (both modulate the HPA axis) (336). Mice deprived of leucine had increased HPA axis activation *via* CRH expression (337). Excessive BCAA concentrations are considered toxic and can cause tissue damage, particularly in the CNS (338). Although exploratory studies remain in their infancy, evidence suggests that BCAA modulation may be useful in cognition disorders (339–341). Further research is required to determine the relationship between the GM, BCAAs, HPA axis, and cognition.

## 4 The relationship between the microbiota, HPA axis, and cognition

### 4.1 Stress-related interactions between the HPA axis and gut microbiota

#### 4.1.1 Evidence from animal studies

The microbiota and HPA axis develop rapidly and profoundly in the first years of life, and environmental stressors can affect both (146–148). Stress experienced during different periods of life can have varying physiological consequences. Early life stressors, and *in utero* stressors, can impact the development and function of the HPA axis (149). Stress during pregnancy disrupts the vertical transmission of microbes from mother to offspring, leading to alterations in the maternal microbiota, which are then transferred to the offspring (342). Jašarević et al. demonstrated changes in the microbiome of these offspring, as well as alterations in the metabolome of the gut and brain (343). In a subsequent study, it was shown that FMT from stressed dams into stress-naïve germ-free mice was sufficient to instill the phenotype observed in stress-exposed offspring (344).

Early life stress, such as maternal separation, activates the HPA axis with associated changes to the developing microbiota, ultimately leading to an imbalance in the GM and an inappropriate stress response (18). Several studies have demonstrated that neonatal stress can lead to short- and long-term alterations in the diversity and composition of the GM (345–347). Interestingly, these consequences appear to be age-dependent. In response to early life stress, younger rats had increased neurogenesis, decreased BDNF IV promoter histone methylation, with a complementary increase in hippocampal BDNF concentration, and associated improvements in spatial and non-spatial learning (348). In contrast, middle-aged rats demonstrated opposing changes, concomitant with impairments in hippocampal-dependent cognitive tasks. These discordant results illustrate the biphasic consequences of early life stress and indicate a role for epigenetic modification of BDNF expression. Furthermore, chronic antidepressant treatment post-exposure was able to rescue the neurological decline observed in the middle-aged rats (348).

A landmark paper by Sudo et al. provided evidence for the essential role of the GM in programming the stress response by illustrating the differences in HPA axis hormones and receptors in germ-free mice when compared to specific pathogen free (SPF) mice (51). Germ-free mice had increased acetylcholine, ACTH, and corticosterone responses following acute stress, indicative of enhanced HPA axis activity. Moreover, these animals showed decreased expression of NMDA receptor subunits (NR-1) in the cortex, and of NR-2a in the cortex and hippocampus, while BDNF levels were lower in the cortex and hippocampus. Following chronic restraint stress, germ-free mice showed significantly greater HPA axis activity, whereas the SPF mice exhibited more anxiety-like behaviors under the same stress (349). These findings have subsequently been reproduced, with both male and female mice demonstrating enhanced stress reactivity to a novel environmental stressors (189, 350, 351). Indeed, germ-free animals show widespread neurodevelopmental changes, associated with alterations in monoaminergic neurotransmission in the CNS (189).

Animal models of chronic stress demonstrate altered intestinal physiology and GM composition, with an increased secretory state and permeability (352–355). Alterations in gut barrier integrity enable bacteria (and microbial-products) to translocate across the mucosa and epithelium, interfacing with immune and neuronal cells (356, 357). Mounting evidence suggests that chronic interactions can lead to systemic, low-grade inflammation contributing to the development of autoimmune, metabolic, and cognitive disorders (358). Moreover, exposure to chronic stress and the subsequent disruption of GM stability has been shown to increase host susceptibility to infection. Mice exposed to prolonged restraint stress demonstrated changes in GM composition, such as bacterial overgrowth and reductions in diversity and richness (359). When challenged orally with the enteric murine pathogen *C. rodentium*, chronically stressed mice had an increased pathogen load and increased colonic TNF-α expression. Probiotics rescued these changes in the GM and the associated host-microbe interactions. A study by Allen et al. demonstrated that the GM is necessary for stress-induced immunomodulation, with enhancement of splenic macrophage reactivity occurring in colonized controls but not in germ-free mice, in response to social disruption stress (360).

The impact of the GM on the HPA axis can further be interrogated following deliberate interventions. Chronic antibiotic treatment led to a decrease in CRH receptor mRNA levels in the brains of rats (361). The introduction of pathogenic bacteria reduced cognitive abilities and heightened anxiety-like behaviors (362, 363). Exposure of neonatal animals to low-dose endotoxins resulted in the activation of TLRs (364, 365). In addition, they showed long-term HPA axis alterations in activity, as evidenced by increased mean glucocorticoid concentrations resulting from an increase in glucocorticoid pulse frequency and amplitude. In an animal model of diet-induced obesity, anxious and depressive-like behaviors were associated with decreased hippocampal levels of glucocorticoid receptors and an exaggerated HPA axis-mediated stress response to acute physical and social stress (366). The probiotic *B. pseudocatenulatum* (CECT 7765) reversed the glucocorticoid receptor and stress response abnormalities, along with the neuro-behavioral phenotype. Probiotic treatment with *Lactobacillus* sp. concurrent with early life maternal separation stress could normalize HPA activity (346). Similarly, pre-treatment with *L. farciminis* reduced HPA hyper-reactivity, intestinal permeability, and neuroinflammation resulting from restraint stress (367). When *L. rhamnosus* was administered to mice, region-dependent alterations in GABA receptor expression in the brain were found to parallel the reduction in stress-induced glucocorticoid levels (191). Notably, the neurochemical effects were not found in vagotomised mice, implicating the vagal pathway. Recently, the GM was shown to influence the expression of genes encoding proteins that participate in the HPA axis and the peripheral metabolism of glucocorticoids (368). A study by Mudd et al. reported a predictive relationship between levels of fecal *Ruminococcus*, serum cortisol, and brain N-acetylaspartate in young pigs (126).

#### 4.1.2 Evidence from human studies

Although there remains a dearth of evidence from human studies, a recent pilot study of 34 healthy infants found that GM composition at one month (measured as alpha diversity) was positively associated with HPA axis reactivity following a painful stressor (369). In a larger cohort of 193 babies (aged 2.5 months), the cortisol stress response was weakly associated with alpha diversity (370). In healthy adults, the experimental administration of LPS in a randomized control trial (RCT) caused a transient physiological stress response, with dose-related increases in cortisol, noradrenaline, body temperature, pulse rate, and cytokines (371). This stress response was associated with increased anxiety and depressed mood. Alterations in cognition occurred both in the short- and long-term, confirming mechanisms for both the promotion and inhibition of cognitive performance during acute inflammatory stress.

In another RCT, a combination probiotic (*L. helveticus* (R0052) and *B. longum* (R0175)) administered to healthy volunteers was associated with beneficial psychological effects in participants and a decrease in 24-hour urinary cortisol, suggesting attenuation of the HPA axis in response to stressors (372). A recent meta-analysis of RCTs focusing on the efficacy of probiotics on stress in healthy individuals showed that probiotic use generally reduced subjective stress levels and appeared to alleviate stress-related sub-threshold anxiety and depression (373). However, cortisol levels were not significantly altered. In a small but detailed four-week study investigating the role of dietary fibre and fermented foods on the GM profile and function, including stress and overall health, subtle GM composition changes were associated with significant changes in several faecal lipids and urinary tryptophan metabolites (374). Participants reported reductions in perceived stress, but these were not significantly different to controls, and markers of stress were unaffected. However, the reduction in perceived stress was dose-dependent, with higher dietary adherence resulting in larger reductions in stress.

### 4.2 HPA axis-related interactions between the gut microbiota and cognition

#### 4.2.1 Cognition

Cognition is defined as the complex mental process of acquiring, understanding, and storing information through thought, experience, and the senses. Essentially, it is the ability to perceive and react, process and understand, store and retrieve, and make decisions and produce appropriate responses. Cognition is not a singular concept, and various ‘domains’ (functions) with several components have been identified. Cognitive dysfunction typically manifests as impairment in one or more aspects of memory, language, visuospatial, execution, computation, understanding, or judgment, amongst others. An increasing body of preclinical and human evidence demonstrates that the MGB axis plays important roles in the development and maintenance of various components of cognition (191, 362, 375).

#### 4.2.2 Evidence from animal studies

Studies in germ-free mice have shown that, in the absence of microbes, the brain is markedly affected, exhibiting deficits in learning, memory formation and recognition, and social and emotional behaviors (58, 123, 124, 362). Gareau et al. demonstrated impaired short-term recognition and working memory in 5 to 6-week-old germ-free mice when compared to conventionally reared counterparts (362). Behavioral differences in germ-free mice include their anxiolytic-like manner when compared to SPF controls, although some studies report increased anxiety-like behavior in different species (124, 189, 351, 376). Microbial colonization has been shown to rescue these elements of cognition, but only if administered during early stages of life (124, 350). Germ-free mice present with social cognitive deficits, which may be associated with biochemical changes, such as decreased hippocampal BDNF and c-FOS expression, important in memory (124, 362, 376, 377). Further studies have demonstrated that reduced cognitive function is inversely associated with BDNF mRNA levels (124, 350).

Several metabolomic studies show alterations in the dopamine and serotonin systems (124, 189, 350, 378–380). These changes include increased hippocampal dopamine D1 receptor mRNA levels and decreased dopamine D1 receptor in the striatum and nucleus accumbens, as well as increased serotonin in the blood and hippocampus, with decreased serotonin receptor expression, respectively.

The role of sexual dimorphism must be noted, as several studies have documented sex-specific differences in the serotonergic system of germ-free animals (350). Female offspring displayed increased cognitive deficits and anxiety-like behavior following prenatal stress, which were associated with GM alterations in the pregnant females and increased IL-1β and decreased BDNF levels *in utero* (381). In contrast, male offspring presented with deficits in social cognition only, without disturbances in the other behavior or cognitive parameters measured (146).

The use of broad-spectrum antibiotics provides another modality to study the cognitive deficits induced by dysbiosis. Administration has been shown to alter tryptophan metabolism and BDNF, NMDA receptor subunit 2B, serotonin transporter, neuropeptide Y, oxytocin, noradrenaline, and vasopressin expression (382, 383). Möhle et al. administered long-term antibiotics to adult mice, resulting in a decrease in hippocampal neurogenesis and memory retention (384). Confirming a role for the GM, these deficits were reversed by a combination of gut flora reconstitution with probiotics and voluntary exercise. The function of the GM was further demonstrated in a recent study comparing antibiotic-treated and germ-free mice with regards to fear extinction learning (385). Without a complex microbiota, both types of mice exhibited altered fear-associated behavior, changes in gene expression in brain cells, and alterations in the firing patterns and rewiring ability of neurons. Selective colonization revealed a critical developmental period, indicating that microbiota-derived signals were required during early post-natal and adult life for normal learning. Given the well-established relationship between glucocorticoids and memory formation, studies like this suggest a complex relationship between the GM, HPA axis and cognitive processes (386).

Animal models can be further used to explore the role of prebiotics and probiotics on cognition and behavior, including changes in depression, anxiety, and stress associated with changes in immune markers, hippocampal synaptic efficacy, and tryptophan metabolism (376, 387). Administering *Mycobacterium vaccae*, a transient commensal microbe, as a probiotic or vaccine to young adult mice improved behavior, learning, and memory during cognitive testing, confirming the role of the immune and serotonergic systems in MGB pathways (388, 389). The probiotic *B. longum* (NCC3001) demonstrated vagal pathway-mediated anxiolytic effects in mice and *B. longum* (1714) showed improved learning and memory after 11 weeks of supplementation (192, 390). Supplementation with various *Lactobacillus* species resulted in improvements in cognitive abilities and social deficits and these changes were shown to occur alongside altered GABA expression in the brain, as well as significant changes in the oxytocin and vagus nerve pathways (191, 391). Six weeks of administration with the probiotic *Clostridium butyricum* restored cognitive function in a mouse model for vascular dementia, which was associated with increased butyrate levels in fecal and brain samples, and activation of the hippocampal BDNF-PI3K/Akt pathway (392).

Infection studies are another useful modality. Administration of *C. rodentium*, combined with acute stress, led to memory dysfunction in young adult germ-free mice (362). This deficit was prevented by daily administration of a *Lactobacillus* sp. probiotic combination before infection. Humann et al. showed that microbial PGs can traverse the mouse placenta, such that maternal administration of PGs could be detected in the fetal brain (64). The offspring exhibited decreased cognitive function related to TLR-2-mediated neuroproliferation *via* FoxG1 induction in the cortex. Neonatal LPS exposure leads to similar cognitive deficits (393). Bilbo et al. revealed that neonatal *E. coli* infection could impair memory in adult rats (394). Intriguingly, the deficit was only observed if an LPS challenge was administered at the time of learning and could be prevented by daily handling of the neonatal rats, which significantly altered their basal HPA axis activity (395).

Animal and human studies indicate an association between prenatal bacterial or viral exposure and the subsequent development of various disorders. Mid-gestation injection of maternal mice with low-dose immunostimulatory polyinosinic:polycytidylic acid (poly I:C) to mimic viral infection significantly impaired non-spatial memory, learning, and motor activity in the offspring at 3 and 9 weeks of age (396). Infection and/or the immune response to mimetics of infectious agents potentially have long-lasting effects on the cognitive abilities of offspring. In humans, the consequences of maternal infection for the microbiota of the offspring, and related cognitive abnormalities, remain unclear and warrant further investigation. These data highlight the interrelatedness of the microbes, the HPA axis, and cognition.

#### 4.2.3 Evidence from human studies

##### 4.2.3.1 Microbial colonization and microbiota development

**Figure 6:**
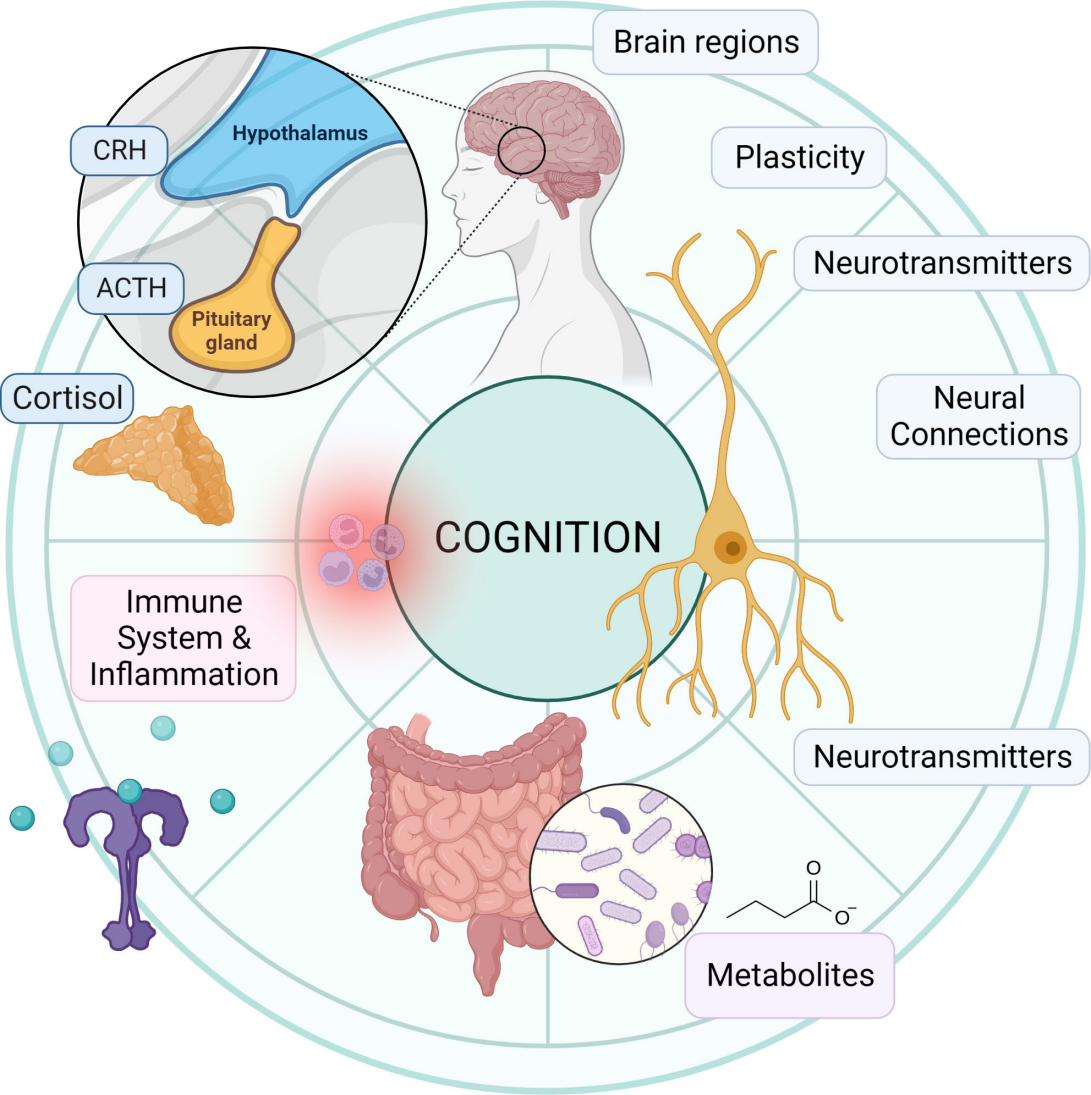
The gut microbiota, HPA axis and cognition. Schematic summary representation of the relationship between the gut microbiota, HPA axis, and cognition. The gut microbiota influences the HPA axis and cognition through the production of metabolites (e.g., SCFAs, and bile acids), neurotransmitters (e.g., serotonin, GABA, catecholamines), and immune system modulation. The HPA axis, comprising the hypothalamus, pituitary gland, and adrenal glands, regulates cortisol release, which in turn affects both gut microbiota and cognitive function. Cognitive processes involve various brain regions (e.g., hippocampus, amygdala, and prefrontal cortex), neurotransmitters, and plasticity, and are modulated by the interplay between the gut microbiota and HPA axis. ACTH, adrenocorticotropic hormone; CRH, corticotrophin-releasing hormone. Figure created with BioRender.com.

Previously, cognition was thought to be regulated solely by the CNS. However, it has become clear that many other non-CNS factors, including the GM, also regulate and influence cognitive function (397). Indeed, it has been postulated that the development of higher cognition in humans may not have evolved in the absence of microbes (398). Considerable attention has recently been paid to unravelling the mechanisms by which the GM, human brain and cognition interrelate (**Figure 6**). Carlson et al. assessed microbiota composition in three groups of one-year-olds, characterized by global and regional brain volume using magnetic resonance imaging (MRI), and cognitive outcome tests, using cluster analysis (399). The group with the greatest abundance of *Bacteroides* outperformed the other two groups using the Mullen Scales of Early Learning assessment. They were also less likely to have been delivered by Caesarean-section. Other studies have also linked delivery mode with neurocognitive development and long-term poor immune and metabolic health outcomes, highlighting the importance of microbial colonization at birth (400, 401). New research suggests that the HPA axis may also be important for linking delivery by C-section to poor health outcomes later in life. In a longitudinal study of 136 infants, those delivered by C-section had lower cortisol concentrations at baseline and in response to a painful stress test at six-month follow-up, signifying an altered HPA axis (402). In a cohort of more than 7,000 neonates, Kiilerich et al. found that levels of inflammatory and stress markers were lower, and growth factor levels higher, in infants delivered *via* pre-labor C-section when compared to those delivered vaginally (403). Interestingly, these differences were not significant if C-section was performed during labor, suggesting that the labor process itself initiates important endocrine, physiological, and biochemical processes relevant to the neonatal immune system and stress response.

##### 4.2.3.2 Antibiotic use

It is well established that the use of antibiotics is critical and necessary when warranted. However, inappropriate use is associated with several negative health outcomes, including the emergence of antimicrobial resistance. Certainly, the MGB axis has multiple sites for off-target activity, which may lead to positive or negative neurocognitive effects downstream, emphasizing the importance of antibiotic stewardship (404–407). Antimicrobial administration in high doses or for long periods can induce severe or irreversible alterations at both the intestinal and brain level (408, 409).

The infant microbiota is highly sensitive to various perturbations like stress and antimicrobials (408). Studies show that perinatal exposure to the various classes of antibiotics substantially alters the establishment of the neonate and infant microbiota, and antimicrobial prophylaxis for C-section delivery is routine. In a RCT of 40 mother/infant pairs in which prophylactic antibiotics were administered before versus after clamping of the cord, the timing of the exposure was found to be critical for microbiota development (410). In a study of over 800 children, antibiotic use in the first year of life was associated with subsequent diminished neurocognitive outcomes (411). Indeed, behavioral difficulties associated with GM composition may persist to at least ten years of age (412). Early-life antibiotic exposure, therefore, appears to disrupt microbiota colonization and maturation, resulting in adverse child health outcomes (413).

Not only infants and children are at risk. In a large prospective cohort of 14,542 participants, chronic antibiotic use in midlife was associated with cognitive impairment later in life, such that antibiotic use was associated with an additional three years of aging, compared to those with no antibiotic exposure (407).

On the other hand, in certain conditions antibiotics may be useful in the treatment of symptoms related to a dysbiotic GM. For example, it is well established that the cognitive impairment arising due to hepatic encephalopathy is mediated by microbial disturbances and can be reversed with oral antibiotic treatment (414–417). Administration of non-absorbable antibiotics in patients with hepatic encephalopathy and mild cognitive impairment improves cognitive performance, subcortical brain activity, fronto-parietal connectivity, and serum metabolomic profile (418, 419).

##### 4.2.3.3 Exogenous glucocorticoids and other drugs

While antibiotics are well known to alter GM composition and function, there are several other types of drugs that influence the GM and may place an individual at risk for cognitive dysfunction (420, 421). Exogenous glucocorticoids are frequently prescribed to treat a multitude of disorders, because of their ability to suppress the immune system and decrease inflammation (422). While we have a cursory understanding of their impact on the GM, and their short- and long-term cognitive effects, a more thorough understanding is critically needed. Several studies have highlighted the deleterious effects of these drugs on cognition (423–428). Both endogenous and exogenous glucocorticoid excess on MR and GR expressed by neurons in the CNS have been shown directly alter the structure and functioning of the brain (429–431). Furthermore, glucocorticoids may modulate the brain indirectly through their effects on the immune system, metabolism, sleep, other hormones, and the GM (432, 433).

Regarding the effects of glucocorticoids on the composition and functioning of the microbiota, some studies have indicated microbiota disruption, while others have shown beneficial effects, which likely depend on the degree of exposure (dose and period) and the type of glucocorticoid studied (434–437) Furthermore, the use of exogenous corticosteroids is not always associated with deleterious effects on cognition, and some studies in animals and humans have failed to demonstrate an association, while some provide evidence of corticosteroid-induced cognitive enhancement (438–443). It appears that the physiological effects of glucocorticoids act in a curvilinear, or ‘inverted U-shaped’, manner on several cognitive systems, where moderate levels are optimal, while very low or high concentrations have distinct adverse cognitive outcomes (444, 445). More research is needed to clarify the deleterious effects and potential usefulness of corticosteroid treatment in MGB-related disorders.

Another group of drugs that has been shown to cause dysbiosis and is associated with cognitive decline is the proton pump inhibitors (PPI) (446, 447). Outcomes vary, with some studies showing no association. Intriguingly, gastric acid suppression by histamine-2 receptor antagonists has more reliably demonstrated associations between chronic use, dysbiosis, and cognitive decline (448).

The gut is often an unwarranted target of chemotherapeutic agents, with mucositis being a common complication (449). Women diagnosed with breast cancer undergoing chemotherapy experience disruption in GM diversity and composition, cognitive impairment, and symptoms of stress, such as anxiety and depression (450).

##### 4.2.3.4 Exposure to Infection

Unsurprisingly, both bacterial and viral infections have been associated with cognitive impairment and functional decline in humans (451–453). *C. difficile* infection is an important example demonstrating these adverse health consequences, beyond acute intestinal dysbiosis (454). Evidence is emerging regarding the potential involvement of *C. difficile* in brain pathologies such as neurodegenerative diseases (e.g. Parkinson’s disease, Alzheimer’s disease), multiple sclerosis, and autism spectrum disorder (455). *C. difficile* has been shown to alter the metabolism of dopamine and interfere with cognitive functions that involve the neuromodulatory action of dopamine, such as motivation and memory consolidation (456). In two case reports in humans, FMT improved gastrointestinal symptoms, cognition, and mood, along with the eradication of *C. difficile* in patients with Alzheimer’s disease (457, 458).

Evidence suggests that the cumulative effect of exposure to multiple infectious pathogens, both bacterial and viral, over several years is associated with changes in cognition, as well as multi-system biological dysfunction before clinical disease is apparent (459, 460). In a large multi-ethnic cohort, an elevated infectious burden, defined as a composite serologic measure of exposure to five specific common pathogens (e.g. cytomegalovirus, *Helicobacter pylori* and herpes simplex virus), was associated with cognitive impairment as assessed by the mini-mental state examination (461, 462). Thus, there appears to be a cumulative effect whereby past infections may contribute to later cognitive impairments. Furthermore, there is extensive evidence in humans showing that prenatal infectious exposure is associated with cognitive and other neurodevelopmental impairments (463, 464).

##### 4.2.3.5 Probiotics and prebiotics

Over time, there has been an increasing recognition of the potential value of probiotics, prebiotics, and combinations thereof. These interventions may be beneficial for patients with dysbiosis, as well as for individuals presenting with other specific complaints or those taking them prophylactically.

Several studies in healthy participants have demonstrated reduced stress-induced cortisol levels and pro-inflammatory cytokines, along with improved cognitive test performance, perceptions of stress, anxiety, and mood (465–469). In a group of healthy women consuming a fermented milk product containing several probiotics, brain activity was found to be enhanced in specific regions associated with cognition (470). In a RCT, multispecies probiotic supplementation protected against the neurocognitive effects of acute stress in healthy women, as measured by neural changes in the frontal cortex using fMRI (471). These findings have been replicated and expanded upon, with studies exploring the relationships between stress, cognition, and the MGB axis. These studies have identified compositional and functional changes in the intestine and brain, along with distinct changes in brain morphology, resting-state brain function, brain activity and functional connectivity in regions known to regulate stress (472, 473). Mechanistic studies have further linked the stress response, cognition, and MGB axis, demonstrating a relative abundance of fibre-degrading bacteria that produce SCFAs, as well as changes in the serotonin and dopamine-norepinephrine pathways (474, 475).

Probiotics have been shown to improve cognitive symptoms and biochemical markers in participants with various dysbiosis-related disorders, including Alzheimer’s disease (476), fibromyalgia (477, 478), and irritable bowel syndrome (IBS) (479). Clinical studies have also suggested that probiotics may decrease anxiety and depression (480). In a study of non-obese and obese individuals, GM composition was found to be associated with scores of cognitive speed, attention, and flexibility, along with significant changes in neural activity in specific brain regions (thalamus, hypothalamus, and amygdala) (481). Co-administration of a probiotic containing *L. rhamnosus*, *B. animalis*, and magnesium chloride for nine weeks to obese male and female participants with depression did not rescue their altered cognition, mood, and intestinal integrity, but did decrease CRP levels (482). Administration of heat-killed *M. vaccae* to terminal lung cancer patients was shown to improve emotional health and cognitive function, thought to be as a result of the enhanced release of neurotransmitters such as serotonin, as part of the immune response to the microbial products (483).

In a study where healthy individuals were given the prebiotic inulin, subjective improvements in mood were noted, along with improved scores on a set of memory tasks (484). In another study, B-GOS supplementation was compared to placebo in healthy participants and found to decrease waking salivary cortisol concentrations and increase the positive processing of information in a dot-probe task (485). Therefore, prebiotics may modulate the HPA axis and benefit cognition.

Barrio et al. reviewed the association between the GM and conditions with neurocognitive impairment, finding that dysbiosis can predict the development of these disorders and influence their pathogenesis (486). Additionally, interventions such as dietary fiber supplementation or probiotics (*Lactobacillus* sp.) have been shown to improve cognitive function and modulate HPA axis activity (reducing cortisol response).

It is important to note that many probiotic trials show no significant effect, especially when performed in healthy populations. For instance, *L. rhamnosus* was not found to be superior to placebo in modifying stress, HPA response, inflammation, or cognitive performance, and the authors highlighted the challenges associated with advancing promising preclinical studies in animals to the clinic (487). Notably, the participants were healthy adult males and not patients with stress-related disorders. A recent systematic review of interventions in children and adolescents to enhance cognitive functioning and emotional behavior found limited consistent effects in these developing populations, although study heterogeneity was seen as a major factor (488). Another systematic review evaluating the utility of prebiotics, probiotics, and fermented food interventions on cognitive performance had negative findings, possibly due to the limited number of small and short-term studies, as well as clinical heterogeneity relating to the population, cognitive tests, and interventions (489). Therefore, further clinical research using adequately powered samples and standardized protocols is warranted.

##### 4.2.3.6 Stress

Chronic stress is associated with various cognitive deficits, systemic inflammation, premature aging, immune system dysfunction, and a higher likelihood of suffering from metabolic disorders such as diabetes, cardiovascular disease, and IBD (490–492). Stress is considered a trigger in patients with functional gastrointestinal disorders, such as IBS and functional dyspepsia, as well as IBDs like ulcerative colitis and Crohn’s disease (493). These conditions exhibit cognitive and behavioral alterations, along with dysbiotic GM profiles.

A study using 3T MRI technology found thalamic volume to be smaller in the IBD group, when compared to healthy controls (494). Yamaoka et al. recently examined the relationship between the GM and stress-related brain functions in healthy subjects, using functional near-infrared spectroscopy (495). They found that the prefrontal cortex stress response correlated with the relative abundance of GM microbes, and that healthy participants with higher stress responses had an increased abundance of microbes found to be associated with depression.

Cognitive alterations may be present in individuals with IBS, and evidence is emerging that specific changes relate to hippocampal-mediated visuospatial memory deficits, which are linked to indices of HPA axis function (20, 496–498). IBS patients also show impairments in tests of cognitive flexibility and have abnormal brain activity in frontal regions of the brain (499).

### 4.3 The relationship between the gut microbiota and cognition, independent of the HPA axis

It is evident that the GM has a significant and profound influence on cognition. Although the HPA axis is centrally placed in linking the various components of the MGB axis with cognitive function, research has demonstrated GM effects on cognition that are independent of the HPA axis. Additionally, while GM signaling pathways and mechanisms involving the vagus nerve, immune system, microbial metabolites, hormones, and neurotransmitters may impact cognition either directly or indirectly through the HPA axis, their involvement might not always require the HPA axis (223).

For example, Gareau et al. demonstrated a lack of memory in germ-free mice in the T-maze test and novel object test in situations with or without stress (362). Frohlich et al. administered antibiotics to adult mice and demonstrated that the resultant cognitive deficits were associated with brain region-specific changes in BDNF, NMDA receptor subunit 2B, serotonin transporter, and NPY (383). Vagus nerve stimulation has been shown to improve cognitive processes and has been attributed to modulation of central noradrenergic and GABA systems, as well as neuronal adaptations within the amygdala, hippocampus, and prefrontal cortex (500). Microbes also create neurotoxic substances such as D-lactate, homocysteine, and ammonia, which can pass through the BBB and affect cognition (321).

Animal models of neurodegenerative diseases have demonstrated associations between the MGB axis and cognition that are likely independent of the HPA axis. In Alzheimer’s disease, CCAAT/enhancer binding protein β/asparagine endopeptidase (C/EBPβ/AEP) signaling mediates disease progression by cleaving both β-amyloid precursor protein and Tau. A recent study demonstrated that gut dysbiosis was positively associated with C/EBPβ/AEP messaging in the brain of a 5xFAD mouse model of Alzheimer’s disease, concomitant with age-related progression of disease severity (501). Chronic antibiotic treatment subdued C/EBPβ/AEP signaling and diminished amyloidogenic processes, rescuing cognitive functions. In a D-galactose/aluminum chloride-induced model of Alzheimer’s disease, selenium nanoparticles-enriched *L. casei* (ATCC 393) administered for thirteen weeks significantly improved cognitive dysfunction and minimized β-amyloid aggregation, hyperphosphorylation of Tau protein, and prevented neuronal death by modulating the Akt/cAMP-response element binding protein/BDNF signaling pathway (502). The intervention was shown to mitigate intestinal barrier dysfunction, inhibit the activation of microglia, and protect brain neurons from oxidative stress and neuroinflammation. Several microbial species can produce amyloid proteins, which can cross the intestinal and BBB when permeable due to inflammation, and promote β-amyloid protein formation and accumulation in the brain, thereby enhancing Alzheimer’s disease pathogenesis in the elderly (503).

In a mouse model that overexpresses the protein α-synuclein, resulting in Parkinsonism, Sampson et al. were able to show that MGB axis signaling is required for motor deficits, microglia activation, and synucleinopathies (75). Oral administration of SCFAs to germ-free α-synuclein mice promoted neuroinflammation and motor dysfunction, and FMT with Parkinson’s disease-affected patients enhanced the Parkinsonism compared to FMT from healthy human donors. In a study by Liu et al., administration of the SCFA butyrate improved cognitive impairments in a mouse model of vascular dementia (392). Huntington’s disease is another neurodegenerative disorder involving psychiatric, cognitive, and motor symptoms. FMT from wild-type mice into a mouse model of Huntington’s disease improved cognitive outcomes, particularly in females (504). Interestingly, simply transplanting the GM of aged rats into young rats induced structural and functional alterations in the brain, as well as behavioral changes indicative of cognitive decline (505).

The influence of diet and dietary habits on the GM is an extensive topic and a primary determinant of GM composition and function. A comprehensive review of this topic is beyond the scope of this review, however, a significant body of evidence demonstrates that consuming a GM-favorable diet can positively influence the GM, improve health status, and enhance cognitive function (506–510). This is likely due to the availability of prebiotics, probiotics, SCFAs, and polyphenols. Conversely, a poor diet and obesity are associated with GM alterations, cognitive dysfunction, and an increased risk for dementia [reviewed in (511)]. Proposed mechanisms include changes in GM composition and function, alterations in microbial metabolites, compromised barrier integrity, and peripheral and central inflammatory processes, as well as hormonal, glucoregulatory, and cardiovascular changes (502, 511, 512).

In obesity, dysbiosis has been associated with cognitive alterations such as hippocampal dysfunction, impaired memory, and reductions in attention and executive function (513). Notably, weight gain and obesity do not appear to be necessary for diet-induced cognitive impairment and age-related cognitive decline (514, 515). Mice colonized with GM from donors fed a high-fat diet demonstrated compositionally distinct microbiota, altered biochemical markers, and developed impairments in exploratory, cognitive, and stereotypical behaviors, in the absence of significant differences in body weight (516). In mice with diet-induced obesity, antibacterial treatment improved metabolic parameters, insulin signaling in the brain, and neurobehavioral changes indicative of anxiety and depression (517). These changes were associated with altered levels of tryptophan, GABA, BDNF, amino acids, and acylcarnitines, while these effects were transferable to germ-free mice by FMT. In this way, FMTs have provided more direct evidence for the role of dysbiotic microbiota in cognitive dysfunction, independent of the HPA axis.

Besides diet, other environmental factors can alter GM composition and heterogeneity, as well as behavioral phenotype and cognition. Jaric et al. showed that even subtle environmental variation, such as the rearing facility of the mice, significantly influenced epigenetic patterns in neuronal genes at the level of chromatin organization, with effects on nucleosome assembly, neuronal differentiation, synaptic plasticity, and regulation of behavior (518). Guo et al. showed that mice exposed to environmental low-dose radiation demonstrated long-term impairments in cognitive function, hippocampal synaptic ultrastructure, and signaling through the Akt/mTOR pathway (519). Indeed, abnormal GM composition may contribute to radiotherapy-induced cognitive decline seen in cancer treatment (520).

## 5 Future perspectives

It is clear that the development and maintenance of higher cognitive function in humans are influenced and modulated by bidirectional HPA axis and GM mechanisms in the complex interplay of the MGB axis. Recognizing these connections opens a plethora of opportunities for novel mechanisms and potential avenues for GM-based diagnostics and therapeutics. While the current evidence is promising, it is essential to acknowledge the need for more research to better understand the underlying mechanisms and the potential for clinical applications in treating conditions with cognitive impairments.

Although GM-based therapeutics are an exciting field and receive much attention, it is vital that rigorous consideration be paid to understanding the mechanistic pathways, as well as the critical window for the development of the GM, HPA axis, and neurological system. Possible therapeutic strategies that could modulate GM and potentially improve cognitive function in different neurological disorders include FMT, prebiotic and probiotic supplementation, and dietary interventions.

While the GM plays an important role in shaping brain function, including the activity of the HPA axis, its role with regards to the distinct structures of the HPA axis remains understudied. Only recently have a small number of studies begun to provide detailed mechanistic insights. Xiang et al. showed that nucleotide-binding oligomerization domain 1 (Nod1) ligands, derived from the GM, modulate catecholamine storage and secretion, and therefore intestinal bacteria modulate the adreno-medullary response through Nod1 sensing in chromaffin cells (521).

Extra-adrenal glucocorticoid production is known to occur in the gut and other tissues, and these glucocorticoids play a highly specific role in regulating local homeostasis, cell development, and immune activation (522–526). However, there is only preliminary evidence available demonstrating that under basal conditions the GM contributes to the regulation of intestinal glucocorticoid production (527, 528).

Furthermore, GM-based studies of adrenal insufficiency and Cushing’s syndrome are also severely lacking, although, as has been demonstrated throughout this review, there are mechanistic reasons to believe that important associations may be uncovered. The most common cause of adrenal insufficiency is autoimmune Addison’s disease, which is thought to be a hapless combination of susceptible genetics and environmental factors. Whether the GM influences the development or pathogenesis of autoimmune Addison’s disease is currently unknown. Many pathogens are known to infect the adrenal cortex, and some of them (e.g., *M. tuberculosis*) can cause adrenal insufficiency (529). A study of chronic adrenal insufficiency patients highlighted that 30% of the participants suffered frequent and incapacitating gut symptoms, consistent with the Rome IV criteria for IBS, and that they had a decreased quality of life (530). However, no evaluation of the GM was performed in this study. Further GM-based research should be prioritized for these two HPA axis conditions.

Finally, a plethora of human diseases have increasingly been associated with changes in the GM. However, whether these changes are causal, consequential, or co-incidental remain unresolved. While animal models have been critical to our understanding of how microbes influence host development and function, and germ-free models are a cornerstone for studying alterations that arise from microbial perturbations, they may not be directly transferable to models of human function. Introducing human microbial communities into germ-free animals may represent a more clinically relevant model. If the field is to move forward, it is necessary that the focus shifts towards determining causal relationships in humans through rigorous and critical approaches and to further advance the translatability of animal and pre-clinical studies to human trials.

In conclusion, this review confirms that the interaction between microbes and the endocrine and neural systems, including the HPA axis, is essential for optimal cognitive function in humans.

## Author contributions

Review written by JR and LD and edited by BL. All authors contributed to the article and approved the submitted version.
